# Pivotal Role of the Chromatin Protein Nupr1 in Kras-Induced Senescence and Transformation

**DOI:** 10.1038/srep17549

**Published:** 2015-11-30

**Authors:** Daniel Grasso, Jennifer Bintz, Gwen Lomberk, Maria Ines Molejon, Celine Loncle, Maria Noé Garcia, Maria Belen Lopez, Raul Urrutia, Juan L. Iovanna

**Affiliations:** 1 Centre de Recherche en Cancérologie de Marseille (CRCM), INSERM U1068, CNRS UMR 7258, Aix-Marseille Université and Institut Paoli-Calmettes, Parc Scientifique et Technologique de Luminy, Marseille, France; 2 Laboratory of Epigenetics and Chromatin Dynamics, Gastroenterology Research Unit, Departments of Biochemistry and Molecular Biology, Biophysics, and Medicine, Mayo Clinic, Rochester, USA

## Abstract

Nupr1 is a chromatin protein, which cooperates with Kras^G12D^ to induce PanIN formation and pancreatic cancer development in mice, though the molecular mechanisms underlying this effect remain to be fully characterized. In the current study, we report that Nupr1 acts as a gene modifier of the effect of Kras^G12D^-induced senescence by regulating *Dnmt1* expression and consequently genome-wide levels of DNA methylation. Congruently, 5-aza-2′-deoxycytydine, a general inhibitor of DNA methylation, reverses the Kras^G12D^-induced PanIN development by promoting senescence. This requirement of Nupr1 expression, however, is not restricted to the pancreas since in lung of Nupr1^–/–^ mice the expression of Kras^G12D^ induces senescence instead of transformation. Therefore, mechanistically this data reveals that epigenetic events, at least at the level of DNA methylation, modulate the functional outcome of common genetic mutations, such as Kras^G12D^, during carcinogenesis. The biomedical relevance of these findings lies in that they support the rational for developing similar therapeutic interventions in human aimed at controlling either the initiation or progression of cancer.

Nupr1, also known as p8 and Com1, is a chromatin remodeling protein originally discovered as a highly inducible gene in the pancreas during the acute phase of pancreatitis and in several tissues in response to numerous pathological stimuli^1^,^2^. Subsequently, studies on Nupr1 gained interest due to its ability to modulate tumorigenesis via the regulation of cell cycle progression, matrix remodeling, autophagy, entosis, apoptosis^3^,^4^,^5^,^6^,^7^,^8^,^9^,^10^,^11^,^12^,^13^ and by activating essential intracellular pathways crucial for pancreatic transformation^14^,^15^. More recently, we reported that in the pancreas of mice genetically engineered to delete Nupr1, the expression of oncogenic *Kras*^*G12D*^ is unable to promote precancerous lesion, PanINs, by an unsuspected mechanism^16^. Thus, in spite of the association of Nupr1 to many pathobiological phenomena, details regarding the molecular mechanisms that contribute to the cancer-associated function of *Kras* have become an area of intensive investigation.

Kras is a small GTPase, which functionally has the ability to induce senescence, proliferation, survival, and apoptosis. The transforming role of oncogenic Kras has been mainly attributed to its promoting effects on cell proliferation and the scape from apoptosis leading to increased cell survival. In contrast, in normal primary cells oncogenic Kras induces a permanent proliferative arrest known as premature senescence^17^. Induction of senescence by Kras is essentially mediated by the upregulation of p16^INK4A^, p19^ARF^, p21^CIP^, p27^Kip1^, Rb and p53 inhibitors of cell proliferation and is thought to serve as a tumor suppressive process which must also be overcome for this oncogene to lead to neoplastic transformation^18^. Noteworthy, the capacity of Kras to induce senescence is depending of the cellular context and the biological setting. For example, the ectopic overexpression of Kras in MEF can trigger senescence, whereas its expression at physiological levels fails to induce the senescent pathway^17^,^19^. In addition, some studies using transgenic mouse models of oncogenic Kras-driven tumorigenesis have documented the presence of senescent preneoplastic lesions in several tissues including pancreas^20^,^21^,^22^. Thus, it remains unclear to what extent the execution of the senescence program is linked to the oncogenic potential of mutated Kras in the pancreas. The only factor that seems to be important to promote the progression of preneoplastic lesions induced by oncogenic Kras into a frank pancreatic cancer is pancreatitis, thought the mediators that are responsible for this phenomenon remains to be fully characterized^23^. Thus, in this context, because Nupr1 is strongly induced in the pancreas with acute pancreatitis^2^ and its role has been associated with cancer, it becomes a good candidate for a molecular that helps the transition from preneoplastic senescent PanIN lesions to a well-established PDAC.

Consequently, the current study was designed to identify mechanisms and molecules involved in this Nupr1-dependent protumoral processes. Fortunately, together, our results demonstrate that Nupr1 regulates *Dnmt1* expression, which in turn generate changes on the genome-wide pattern of DNA methylation that are required for transformation after oncogenic activation. The pathobiological relevance of these mechanistic insights is underscored by the fact that tumor development was prevented in Kras^*G12D*^ expressing mice treated with an inhibitor of DNA methylation. Altogether, the data reported here support the early implication of a genetic-to-epigenetic crosstalk as a novel mechanism underlying tumor development and highlight the opportunities that pharmacologically interfering with these events offer to the potential treatment of this malignancy.

## Results

### Genetic inactivation of Nupr1 results in the downregulation of *Dnmt1* expression with accompanying genome-wide changes in DNA methylation

**Figure 1:**
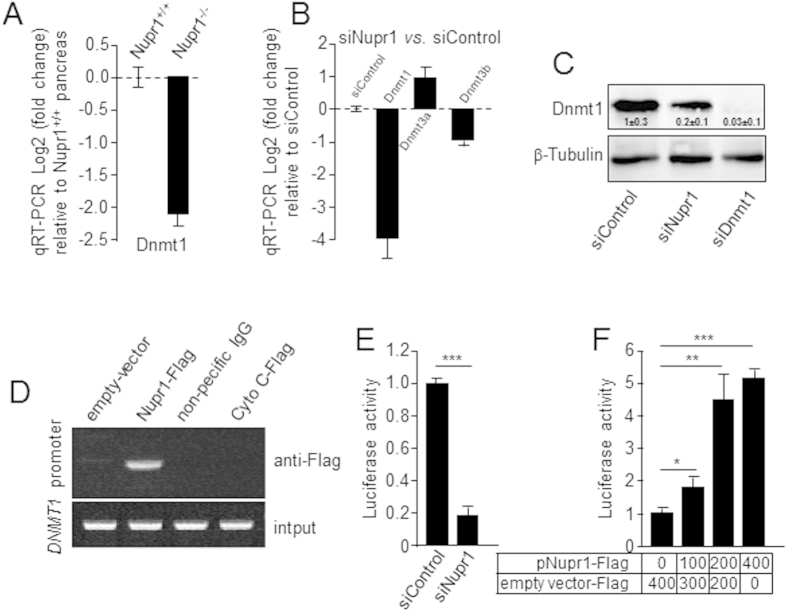
Nupr1 depletion reduces *Dnmt1* expression. **(A)** RT-qPCR showing decrease of Dnmt1 transcript in Kras^*G12D*^ expressing mouse pancreas Nupr1^–/–^ (n = 3) compared to Nupr1^+/+^ (n = 3). **(B)** By RT-qPCR, little modification in Dnmt3a and Dnmt3b but important Dnmt1 decrease is observed in siNupr1-treated MiaPaCa2 cells. **(C)** MiaPaCa2 cells were transfected with siNupr1 or siDNMT1 and expression of DNMT1 was measured by western blotting **(D)** MiaPaCa2 cells were transfected with pCDNA3 vectors containing a Flag-tagged DNMT1, an irrelevant Cytochrome C or an empty vector (Empty). ChIP was performed using an anti-Flag antibody or a non-relevant IgG. (Top) Occupancy of Nupr1 on Dnmt1 promoter, (bottom) DNA input (10%). **(E)** Cells were transfected with combinations of a Dnmt1 promoter-Luc vector with siControl or siNupr1, and pSV40-RL as an internal control. After 48 h, luciferase activity was determined and expressed as the ratio of specific luciferase activity/internal standard. **(F)** Cells were transfected with combinations of a Dnmt1 promoter-Luc vector with increasing amounts of pNupr1-Flag construct, and pSV40-RL as an internal control. After 24 h, luciferase activity was determined and expressed as the ratio of specific luciferase activity/internal standard. Means ± SD; *p < 0.05, **p < 0.01***p < 0.001.

We evaluated whether key epigenetic regulators were differentially regulated in the pancreas of *Kras*^*G12D*^; Nupr1^+/+^ *vs*. *Kras*^*G12D*^; Nupr1^–/–^ mice at 5 week after birth, a stage preceding the appearance of PanINs, by generating and analyzing genome-wide expression profiles (accession number GSE45232), which lead to the observation that DNMT is downregulated under this conditions. Indeed, using quantitative RT-PCR, we found that Nupr1 inactivation decreased *Dnmt1* expression in the pancreas tissue by 2.1 ± 0.3 folds (*p* < 0.01) (Fig. 1A). Complementary knockdown experiments using 2 specific Nupr1 siRNAs to transfect pancreatic cancer cells (MiaPaCa2) decreased Dnmt1 mRNA levels (3.8 fold ± 0.7; *p* < 0.01), without affecting either Dnmt3a or Dnmt3b expression (Fig. 1B). The, downregulation of Dnmt1 at the protein level was confirmed using western blot analyses (Fig. 1C). ChIP assays demonstrated that Nupr1 binds to the *Dnmt1* promoter, revealing a direct effect for this protein in the transcriptional regulation of this methylase (Fig. 1D). Luciferase-based gene reporter assay in MiaPaCa2 cells transfected with siNupr1 or siControl demonstrated that the activation of the *Dnmt1* promoter is dependent on the expression of this protein (Fig. 1E) since the reporter activity decrease from 1.0 ± 0.2 to 0.2 ± 0.02; (*p* < 0.001) when Nupr1 was knockdown. Conversely, overexpression of Nupr1 increased *Dnmt1* promoter activity in a dose-dependent manner as showed in Fig. 1F. The increase in the reporter activity was of 1.8 ± 0.3, 4.3 ± 0.9 and 5.1 ± 0.3 folds with 100, 200 and 400 ng of pNupr1-Flag construct. Similar results were obtained using another pancreatic cancer cells, Panc1 (supplemental Figure 1). Together, these results suggest that expression of Nurp1 can impact on DNA methylation. To test the validity of this idea, we performed a genome-wide MeDIP-based 5-methyl-cytosine analysis on DNA samples derived from MiaPaCa2 pancreatic cancer cells treated with either siNupr1 or siControl. Methylated DNA was first enriched using 5-methyl cytosine antibody-based capture method, labeled with C3 and C5 and hybridized to NimbleGen Human DNA Methylation 3 × 720 K CpG Island Plus RefSeq Promoter Arrays. Methylation data was generated using C3 *vs*. C5 subtractive image processing with peak calling as previously described^24^. The results of these experiments, shown in both supplemental Fig. 2 and supplemental Table 1, demonstrate that Nupr1 downregulation clearly modifies the DNA methylation status in pancreatic cancer cells. Depletion of Nupr1 resulted in 877 newly methylated genes and demethylation of 2026 genes (supplemental Figure 2 and supplemental Table 1). To validate the functional consequences of this epigenetic change, we used RT-qPCR to measure the changes in expression of a subset of mRNA encoded by the hypomethylated genes. The results showed in supplemental Fig. 3 demonstrate that 8 out of 10 methylated genes become downregulated. Ontological analysis revealed a modification pattern of genes which functions are primarily related to histone modification and chromatin organization, signaling, cell proliferation, cell adhesion and motility. Regarding cell signaling cascades, pathway enrichment analyses show that Nupr1 depletion induces methylation of genes related to the JAK-STAT signaling and the EGF pathway, while causing demethylation of genes related to Wnt pathway and Fork head family. These results demonstrate, that consistent with its effects on Dnmt1 expression, the genetic inactivation of Nupr1 gives rise to changes in genome-wide methylation events that affect various gene expression networks.

### Nupr1 deficiency on MEF expressing Kras^*G12D*^ induce senescence instead transformation

**Figure 2:**
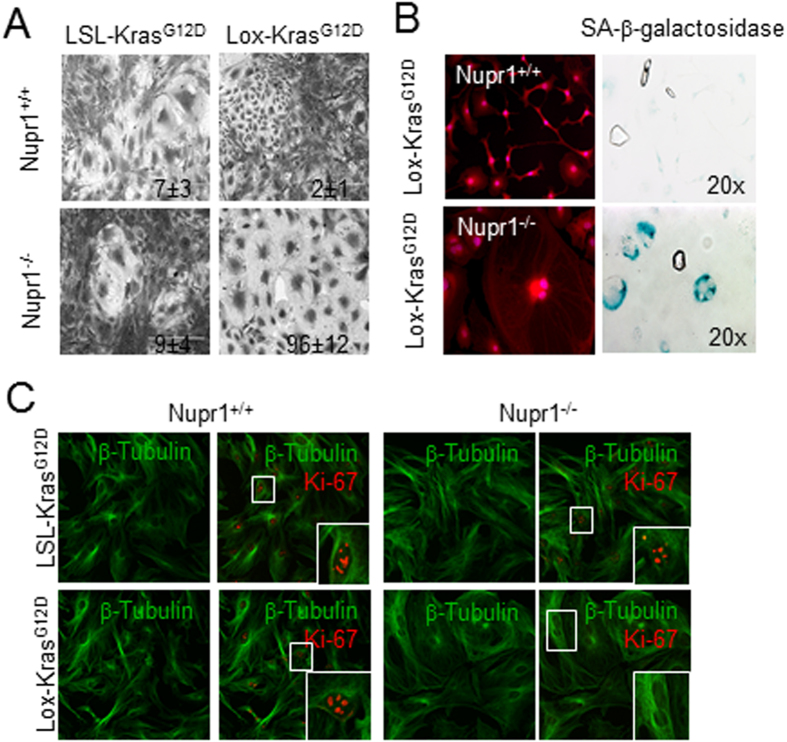
Expression of Nupr1 switches OIS to transformation in MEFs. (**A,B**) MEF from LSL-Kras^G12D^; Nupr1^+/+^ and LSL-Kras^G12D^; Nupr1^–/–^ mice were obtained and transduced with an adenovirus expressing the Cre recombinase. Transformation-like images were observed in Nupr1^+/+^ cells whereas pictures compatible with senescence were observed in the Nupr1-deficients cells. Values correspond to the percentage of senescent cells. **(A)** and a strong SA-βGal staining was observed in Nupr1^–/–^ MEFS whereas no activity was detected in the Nupr1 wild-type cells **(B)**. Tubulin and Ki-67 staining indicates a rearrangement of the cytoskeleton compatible with a senescent phenotype combined with not proliferation in Nupr1-deficients MEFs **(C)**.

To gain insight into the cell biological phenomenon which may results from these genomic changes, we subsequently studied the cellular effect of oncogenic *Kras* activation using MEFs derived from Nupr1^+/+^; LSL-Kras^*G12D*^ and Nupr1^–/–^; LSL-Kras^*G12D*^ mice and transduced them with Cre-expressing adenovirus to activate Kras^*G12D*^. Phenotypically, Nupr1^+/+^; Lox-Kras^*G12D*^ MEFs adopt the morphology previously described for oncogene-transformed fibroblasts and increased growth capacity (Ki-67 positive nuclei = 9.4 ± 1.2 per field) (Fig. 2C) as previously described^19^. In contrast, MEFs from Nupr1^–/–^; Lox-Kras^*G12D*^ mice displayed an elongated morphology with low Ki-67 staining (Ki-67 positive nuclei = 0.5 ± 0.4 per field), features reminiscent of senescent cells (Fig. 2A,C). Finally, senescence associated (SA)-βGAL staining was positive in Nupr1^–/–^; Lox-Kras^*G12D*^ MEFs but not in control cells as showed in Fig. 2B. Together these data demonstrate that, at the molecular level, the inactivation of Nupr1 changes DNA methylation genome-wide, while at the cellular level, it promotes Kras-induced senescence.

### Enhanced *Dnmt1* expression counteracts the effect of Nupr1 deficiency on Kras^*G12D*^ induced senescence in cultured pancreatic cancer cells

**Figure 3:**
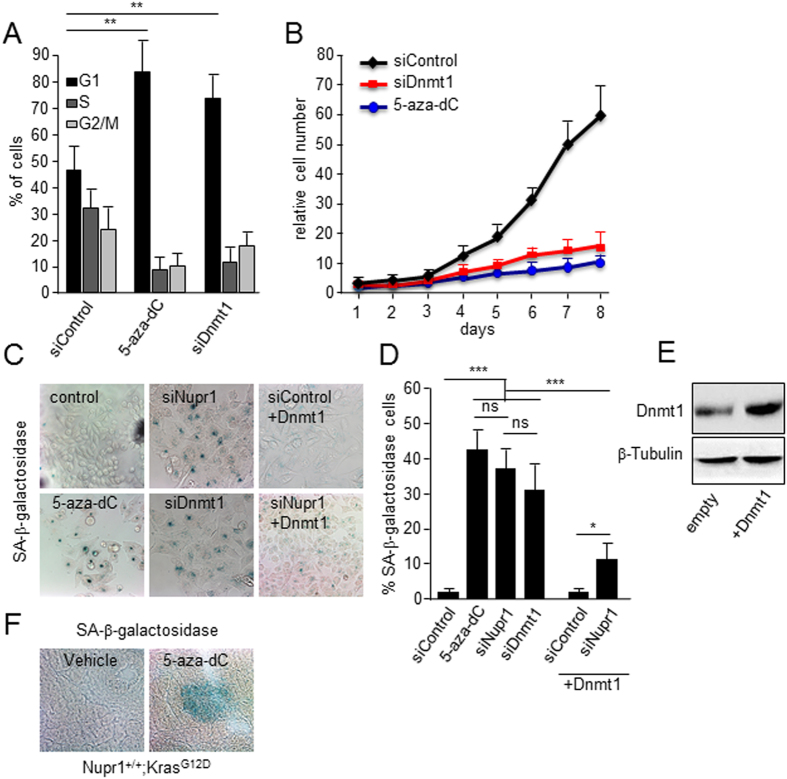
DNA hypomethylation triggers OIS in pancreatic cancer cells. **(A)** Arrest in cell cycle and accumulation of G1 cells is observed in 5-aza-dC and siDnmt1 treated cells. **(B)** Proliferation curve showing that either 5-aza-dC treatment or siRNA-mediated Dnmt1 silencing stops cell proliferation. The relative cell number at each time point on the growth curves represents the means value ± SD of triplicates normalized to the cell number at day 1. **(C)** SA-βGal activity staining in MiaPaCa2 cells. As with siNupr1 treatment (upper middle panel), 5-aza-dC (lower left panel) and siDnmt1 (lower middle panel) treatments induce cells enter in senescence. On the other hand, the constitutive overexpression of Dnmt1 in MiaPaCa2 cells significantly rescues the siNupr1-induced senescence (lower right panel and right plot). **(D)** quantification of SA-βGal staining. **(E)** Expression of DNMT1 in MiaPaCa2 transfected with empty and pCMV6-Dnmt1 plasmid **(F)** SA-βGal staining in pancreas from Nupr1^+/+^ Kras^*G12D*^-expressing animals treated with 5-aza-dC (n = 6) or vehicle (n = 6). Means ± SD; ns = no significant, *p < 0.05, **p < 0.01, ***p < 0.001. Scale bar, 20 μm.

We first treated MiaPaCa2 cells with 1 μM 5-aza-2′-deoxycytydine (5-aza-dC), a general inhibitor of DNA methylation. Figure 3 show that 5-aza-dC induced cell cycle arrest (mean 81 ± 11% of cells in G1 vs. 46 ± 8 in control cells; *p* < 0.01) as judged by the standard propidium iodide staining protocol followed by flow cytometry analysis (Fig. 3A); inhibits cell proliferation (mean 62 ± 9 folds increase in control cells vs. 8 ± 1 in 5-aza-dC treated cells after 8 days of growth; *p* < 0.001) (Fig. 3B); and become positive for SA-βGal staining (mean 42% ± 8 *vs.* 2% ± 1 of siControl; *p* < 0.001) (Fig. 3C,D). Altogether, these results demonstrate that drug-induced DNA demethylation triggers cell growth arrest and cellular senescence. Similarly, knock down of Dnmt1 using specific siRNAs induces senescence in these cells (SA-βGal mean 29% ± 7 *vs.* 2% ± 1; *p* < 0.001) (Fig. 3C,D). We also performed senescence assays in MiaPaCa2 cells that stably overexpresses Dnmt1 but had either decreased or wild-type levels of Nurp1. We find that the downregulation in Nurp1 levels induces senescence in a manner that can be antagonized by overexpressing Dnmt1 (37% ± 7 *vs.* 11% ± 3 of Dnmt1 overexpressing cells; *p* < 0.01) as shown in Fig. 3C,D. We then validate this hypothesis *in vivo* by showing that SA-βGal staining was readily detected in these pancreata from Nupr1^+/+^; Kras^G12D^ treated with 5-aza-dC but not in untreated pancreas (Fig. 3F). Morphometric analysis reveals that mice treated with 5-aza-dC display a 13 ± 4% of their area stained with SA-βGal whereas in the vehicle-treated pancreas this staining occupied 2.5 ± 2% (*p* < 0.01; n = 6) of the total pancreas sections. Thus, combined, the data from pharmacological, RNAi knockdown, and overexpression experiments are congruent in revealing that Nupr1 maintains a genome-wide level and pattern of Dnmt1-mediated DNA methylation that is necessary to counteract the induction of Kras^G12D^-induced senescence. This result are consistent, but more importantly, extend previous studies showing that the knockout of Nurp1 inhibits Kras^G12D^-induced PanINs formation, which is known to require a bypass of oncogene-mediated senescence^16^.

### Drug-induced inhibition of DNA methylation prevents PanIN development in Kras^*G12D*^ expressing mice

**Figure 4:**
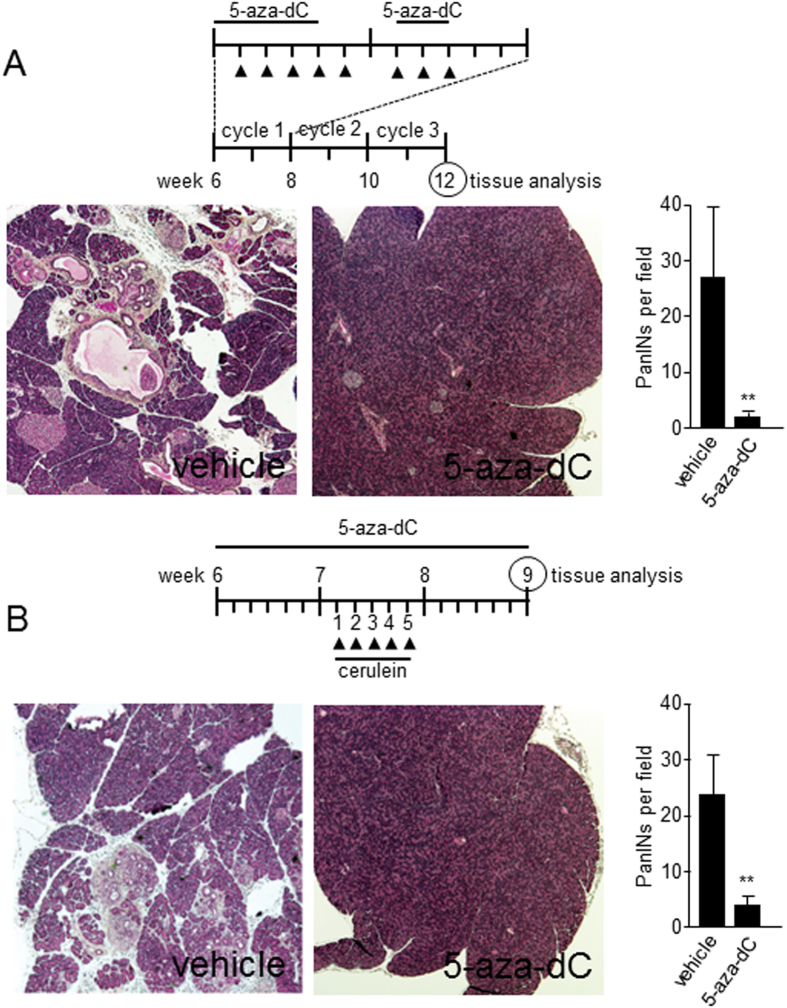
Inhibition of DNA methylation prevents PanINs development. **(A)** 6 weeks old Nupr1^+/+^ Kras^*G12D*^ expressing mice were treated with the inhibitor of DNA methylation 5-aza-dC during three cycles of two weeks as represented by the scheme. Several PanINs lesions were found in pancreas tissue from vehicle-treated animals in contrast to 5-aza-dC-treated animals where almost no lesion was detected. **(B)** Nupr1^+/+^ Kras^*G12D*^ expressing mice were submitted to a cerulein-induced pancreatitis treatment to increases the oncogenic Kras transforming pressure. Compared to vehicle-treated mice, pancreatic from DNA methylation inhibitor-treated animals were resistant to PanINs development with a significant reduction in PanINs development. Means ± SD; **p < 0.01.

Based on the fact that inactivation of Nupr1 impairs Kras^*G12D*^-associated changes in DNA methylation to establish senescence (oncogene-induced senescence, OIS) through downregulation of Dnmt1, we hypothesized that treatment of Kras^*G12D*^ expressing Nupr1^+/+^ animals with inhibitors of DNA methylation should mimic this effect. To explore this concept, we randomized 12 Kras^*G12D*^ expressing Nupr1^+/+^ mice into two groups. One of these groups was treated with the DNA methyltransferase inhibitor 5-aza-dC (24 injections of 250 μg/kg of body weight following the protocol described in Fig. 4A) while the control group received vehicle alone. Figure 4 demonstrates that treatment with 5-aza-dC significantly reduces Kras^*G12D*^ induced development of PanIN lesions (2 ± 1 vs. 27 ± 17 lesions; *p* < 0.005; n = 6) (Fig. 4A). Moreover, since oncogenic mutations in KRAS cooperate with pancreatitis to give rise to pancreatic cancer with high penetrance, we also investigated whether treatment with 5-aza-dC prevents pancreatitis-enhanced PanIN development. Accordingly, 6 week-old Kras^*G12D*^ expressing mice, which carry wild type Nupr1 alleles, were treated with either 5-aza-dC or vehicle for 3 weeks. At the end of the first week of 5-aza-dC treatment, mice were also injected with cerulein for 5 days (Fig. 4B). Through these experiments, we found that 5-aza-dC-treated mice were resistant to cerulein-enhanced PanIN induction (3 ± 3 vs. 23 ± 9 lesions in 5-aza-dC and vehicle groups respectively, *p* < 0.005; n = 6) (Fig. 4B). Thus, pharmacological inhibition of DNA methylation can antagonize the formation of PanINs under both, basal or cerulein-stimulated conditions.

### Genetic inactivation of Nupr1 results in switching transformation to oncogene induced senescence in Kras^G12D^-induced lung adenomas

**Figure 5:**
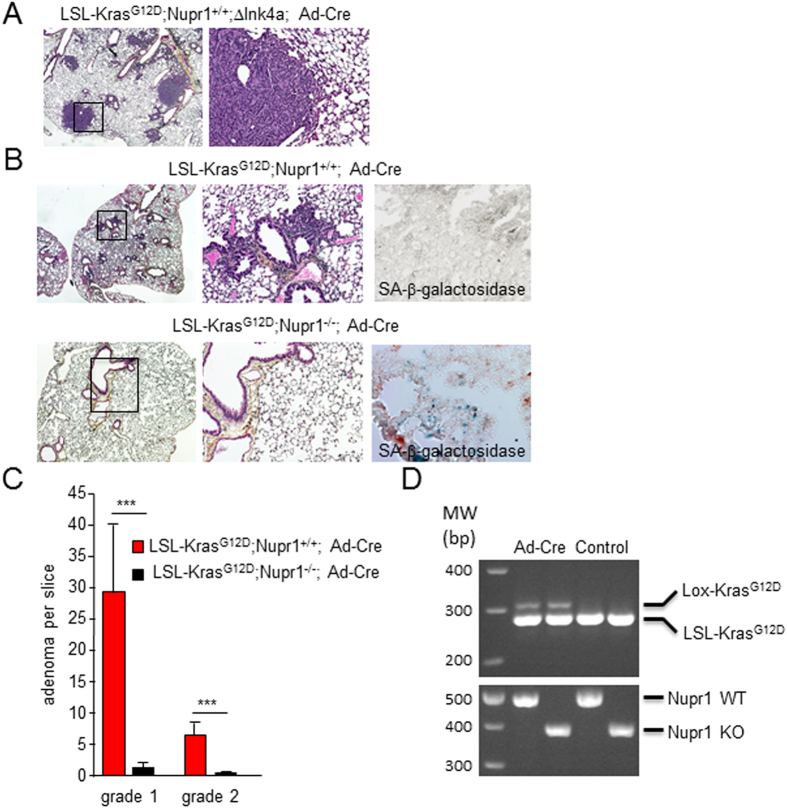
Nupr1 expression is necessary for transformation induced by the oncogenic Kras in lung. **(A)** LSL-Kras^G12D^; Nupr1^+/+^; ΔInk4a mice were treated by intratracheal administration of the adenovirus expressing the Cre recombinase and 12 weeks later the lungs were processed for H&E analysis. All animals showed adenocarcinomas indicating the efficient recombination. **(B)** LSL-Kras^G12D^; Nupr1^+/+^ and LSL-Kras^G12D^; Nupr1^–/–^ mice were treated with the adenovirus expressing the Cre recombinase as in A and lung processed for H&E analysis and SA-βGal staining. Mice with Nupr1^+/+^ background show several adenoma lesions and negative SA-βGal staining whereas on the contrary lung from mice with Nupr1^–/–^ background present almost not adenoma with positive SA-βGal staining. **(C)** Quantification of adenoma grade 1 and grade 2. Means ± SD; ***p < 0.001. **(D)** Lox (size 325 bp) and LSL (size 287 bp) *Kras*^*G12D*^ and Nupr1 KO (size 381 bp) or wt (size 489 bp) were amplified.

Deletion of the *Nupr1* gene in mice prevents Kras-induced PanIN development^14^ and PDAC in around 50% of animals^25^ by blocking the oncogenic Kras-dependent RelB and IER3 intracellular pathway on one hand^15^ and, on other hand, by regulating the role of oncogenic Kras^G12D^ by an unknown mechanism^16^. Whether or not this effect is limited to the pancreatic tissue remained still unresolved. To address this issue, we first utilized the LSL-Kras^G12D^; ΔInk4a mice for validating Kras activation after intra-tracheal administration of the Ad-Cre adenovirus and approach which, after 12 weeks gave rise to lung adenocarcinoma (Fig. 5A). After this validation step, we administered the same treatment to the LSL-Kras^G12D^ mice, carrying wild-type Nupr1 alleles or knockout. In this experiments, Nupr1^+/+^ developed a significant number of adenomas grade 1 and 2 (29.1 ± 11.3 and 6.5 ± 2.3 per slice) while Nupr1^–/–^ mice developed fewer lesions (1.5 ± 1.0 and 0.1 ± 0.1 lesions) (Fig. 5B,C). Most importantly, whereas a significant SA-βGal activity was found in Nupr1^–/–^ mice staining for this enzyme was no detected in Nupr1^+/+^ animals (Fig. 5B). Efficient recombination after Ad-Cre was validated in both Nupr1^+/+^ and Nupr1^–/–^ lungs (Fig. 5D). In conclusion, in both, pancreas and lung, expression of Nupr1 seems necessary to switch Kras^G12D^-induced senescence to transformation.

## Discussion

A nascent set of critical studies indicates that epigenetic mechanisms, which can both silence and activate genes in an inheritable manner independently of the coding capacity of DNA, play a significant role in PDAC. The discovery of these epigenetic events that determine the outcome of this malignancy is important since, contrary to genetic changes, these molecular mechanisms are largely reversible using small drugs. Therefore, targeting of epigenetic regulators is a promising to developing new therapeutic modalities to treat pancreatic cancer. Moreover, epigenetic markers have begun to inform the diagnosis and prognosis of many malignancies, and as they continue to be discovered, will likely soon be applicable to the management of pancreatic cancer. Congruent with this line of research, in this work, we demonstrate that the chromatin binding protein, Nupr1, is a key molecule responsible for the crosstalk between genetic and epigenetic mechanisms that, by acting in concert, facilitate the progression of pancreatic cancer. Thus, it becomes important to discuss how our results contribute to extend the current knowledge in this field of pancreatic cancer research. For instance, key findings reported here demonstrate that Nupr1 acts as a modifier of Kras-induced senescence, a phenomenon that antagonizes transformation by this oncogene. A search for mechanisms underlying this phenomenon revealed that Nupr1 regulates Dnmt1 expression which in turn induces global changes in DNA methylation. This phenomenon leads to the changes in the methylation status of genes primarily involved in cell cycle progression and other associated to specialized functions characteristic of the normal cell phenotype. Thus, we believe that these findings suggest that Nupr1 is involved in maintaining a specific DNA methylation pattern that facilitates transformation, a function that is inactivated through the genetic inactivation of this protein. Hence, this new information has clear mechanistic relevance for understanding how chromatin proteins such as NUPR1 and DNMT1 work downstream of the Kras oncogene to promote neoplastic transformation in the pancreas.

The results of this study conducted in the pancreas led us to search for whether they have a more general application to other system. Careful review of previous studies revealed that Gazin *et al.* pioneered the finding that epigenetic changes are necessary for the transformation of NIH3T3 cell by mutant Kras, including the regulation of Dnmt1 expression in a 5-aza-dC-sensitive manner^26^. Other investigators have also reported that epigenetic changes are not restricted to Kras-induced neoplastic transformation, but rather other oncogenes also require changes in DNA methylation (reviewed in^27^,^28^,^29^) to perform their function. These data are important to consider since the cancer promoting function of Nupr1, though highly studied in the pancreas and in relationship to its role as modifier of Kras function, is neither exclusive for this organ nor for this oncogene.

Moreover, we tested whether these observations have potential biomedical relevance for the treatment of pancreatic cancer through an experimental therapeutic trial in genetically engineered mice using a pharmacological Dnmt1 inhibitor. Our results using this drug showed that they can increase Kras-induced senescence with a concomitant reduction in PanIN formation thereby lending additional support to the notion that DNA methylation is at the core of a crosstalk between Nupr1 and Kras. In addition, these experiments reinforce the concept that epigenetic changes are *bona fide* targets for treating pancreatic cancer development. Indeed, these types of inhibitors have been considered as part of the arsenal of chemotherapeutic agents for the treatment of many malignancies, including those that originate in the pancreas^30^. Unfortunately, however, these drugs belong to the first generation of Dnmt inhibitors which are not optimal for the treatment of common cancers because they are toxic to normal cells at standard high doses. Fortunately, more recently studies have demonstrated that low doses of these inhibitors, which seem to be non-toxic for patients, are able to reprogram cancer cells to a more normal phenotype^31^. Using the currently available drugs, as well as the possibility of developing new inhibitors with lower toxicity, thus builds the trajectory toward the design of useful protocols that apply these drugs to the treatment of patients with advanced pancreatic cancer. Alternatively, either low dose protocols and combination therapy with currently available Dnmt1 inhibitors or less toxic inhibitors may be more appropriate as chemopreventive agents in high risk patients who carry preneoplastic pancreatic lesions, such as PanIN or premalignant cystic tumors. Nevertheless, our studies provide a rationale, based on mechanistic evidence, for further designing and exploring these modalities for the treatment of this deadly pancreatic malignancy.

In summary, when combined, the results of the experiments reported in this paper support the notion that epigenetic changes mediated by chromatin proteins, such as Nupr1, along with their effects on DNA methylation modulate the function of well-characterized oncogenes, which mutational activation occurs early during the development of cancers. The finding that modulation of epigenetic changes can antagonize the function of these powerful oncogenic mutations offer a less gene-centric vision of pancreatic cancer thus helping to expand our mechanistic understanding of this disease. In light of the reduced success of previous attempts to correct genetic alterations in pancreatic cancer through gene therapy, these considerations also highlight the possibility that early intervention against epigenetic changes may be more beneficial for the management of this cancer and likely other malignancies, which are known to associate with Kras mutations.

## Material and Methods

### Animals

*Nupr1*^*–/–*^ mice bear a homozygous deletion of exon 2 of the *Nupr1* gene and were reported previously^32^. The *Pdx1-cre; LSL-Kras*^*G12D*^ mice were provided by R. Depinho (Dana-Faber Cancer Institute, Boston, Massachusetts, USA) and resulted from crossbreeding of the following strains: *Pdx1-Cre*^7^ and *LSL-Kras*^*G12D*^^33^. For DNA methylation inhibition we used i.p. injections of 5-aza-2′-deoxycytidine (Sigma) at 250 μg/Kg of body weight. Pancreatitis was induced by cerulein (Sigma) i.p. administration at 250 μg/Kg of body weight five consecutive days followed by one week of recovery. Because animals are from different genetic backgrounds, we systematically used littermate control and experimental mice. Mice were kept within the Experimental Animal House of the *Centre de Cancérologie de Marseille* (CRCM) pole Luminy. All experimental protocols were carried out in accordance with nationally approved guidelines for the treatment of laboratory animals. All experimental procedures on animals were approved by the Comité d’éthique de Marseille numéro 14.

### Methylated DNA immunoprecipitation (MeDIP), amplification and DNA methylation CpG island promoter arrays

High-quality genomic DNA was extracted from MiaPaCa2 cells with siControl or siNupr1 utilizing the phenol:chloroform:isoamyl alcohol method. DNA was sheared to generate fragments between 100–600 bp by sonication via the Bioruptor® XL (Diagnode). MeDIP was performed on the sheared DNA using the MagMeDIP kit according to manufacturer’s instructions (Diagenode). Input and immunoprecipitated DNA was purified (Qiagen) and quantified using NanoDrop (Thermo Scientific). Whole-genome amplification for both input and MeDIP DNA, hybridization to NimbleGen Human DNA Methylation 3 × 720 K CpG Island Plus RefSeq Promoter Arrays, and raw data processing was conducted by Ambry Genetics. Gene list clustering was performed using the DAVID Bioinformatics Resources 6.7 software (NIAID, NIH)^34^,^35^.

### Cell culture

MiaPaCa2 and Panc1 cells obtained from ATCC (CRL-1420) were maintained in DMEM (Invitrogen) supplemented with 10% FBS at 37 °C with 5% CO_2_. To produce Dnmt1 stably expressing cells, human Dnmt1 cDNA was cloned into pCMV6-Myc-DDK vector. The pCMV6-Dnmt1 plasmid was transfected with Lipofectamine 2000 reagent (Invitrogen) in MiaPaCa2 cells and selected for three weeks with G418 (Geneticin; Invitrogen). Dnmt1 expression was checked by western blot. INTERFERin reagent (Polyplus-transfection) was used to perform siRNA transfections according to the manufacturer’s protocol. Scrambled siRNA targeting no known gene sequence was used as negative control. The sequences of Nupr1-specific siRNA [siNupr1 r(GGAGGACCCAGGACAGGAU)dTdT and siNupr1#2 r(AGGUCGCACCAAGAGAGAA)dTdT]^36^, and Dnmt1 siRNA [siDnmt1 r(GGAAGAAGAGUUACUAUAA)dTdT]^37^ were previously reported and were from Qiagen. For general DNA methyltransferases inhibition cells were incubated with 1 μM 5-aza-2′-deoxycytidine (Sigma). Cell cycle analysis was performed by standard propidium iodide staining protocol on a FACSCalibur flow cytometer (BD Biosciences). Data analysis was performed using CellQuest (BD Biosciences) or FlowJO (Treestar) software.

### MEFs transduction

MEFs from LSL-Kras^G12D^; Nupr1^+/+^ and LSL-Kras^G12D^; Nupr1^–/–^ mice were derived from E14 embryos following standard protocols^11^ and grown in DMEM media supplemented with 10% FBS, 2% glutamine, 1% non-essential amino acids and 1% β-mercaptoethanol. For transduction experiments, MEFs were seeded at 10,000/well in 12-well plates. After 24 hours, the medium was replaced with DMEM containing 1% FBS, and the cells were infected with 5 MOI of Ad-Cre adenovirus supplemented with 1 μg of Polybrene per ml. On the next morning, the supernatant was replaced with fresh medium containing 10% FBS. The Ad-GFP, expressing GFP, was used in the same conditions as control of transduction efficiency. Under these condition 100% of the cells became transduced by the adenoviruses.

### Histology and H&S staining

Pancreatic and lung sections were fixed in 4% paraformaldehyde and paraffin embedded. H&E staining was performed using standard procedures. Sections were examined in an Eclipse 90i Nikon microscope.

### SA-β-galactosidase activity

Pancreas and lung cryosections from mice or cells cultured on glass coverslips were tested for SA-βGal activity using the Senescence β-galactosidase Staining Kit (Cell Signaling) according to the manufacturer’s protocol.

### RT-qPCR

Pancreas RNAs from Nupr1^-/-^ and Nupr^+/+^ Kras^*G12D*^mice were prepared immediately after dissection following Chirwin’s protocol^38^. RNA from cells was prepared using Trizol Reagent (Invitrogen) and reversed transcribed using Go Script (Promega) according to manufacturer’s instructions. RNA from MiaPaCa2 cells treated with siControl and siNupr1 was prepared as previously reported^39^ and expression of GLTSCR2, JAG2, HABP4, CREB5, DLX3, FAT4, CPSF1, TPM4, TOB1 and RNF4 mRNA by RT-qPCR was performed by RT-qPCR using a Stratagene cycler and Takara reagents, according to the manufacture’s instruction. Primers sequences are available upon request.

### Chromatin immunoprecipitation (ChIP) assay

MiaPaCa2 cells were transfected with either the Nupr1-Flag, irrelevant cytochrome C-Flag or empty vector-Flag constructs and, subsequently, ChIP assay performed using the EZ-ChIP kit (Millipore) according to the manufacturer’s instructions. Input DNA was collected after pre-clearing lysate with Protein G/agarose beads prior to immunoprecipitation with the anti-FLAG (M2) antibody (Sigma) or with a non-relevant IgG. PCR was performed using TaKaRa LA Taq with GC Buffer II, according to the manufacturer’s suggestions (Takara Bio Inc). A 212-bp region of the Dnmt1 gene promoter was amplified by PCR, using specific primers (5′-CACTGGCCGTCCCGGCCATCTC-3′, 5′-GCACCGTTCTCCAAGGACAAATC-3′).

### Luciferase assay

MiaPaCa2 and Panc1 cells were plated at 70% confluence in six-well plates and 24 h later transiently transfected with 3 μg total DNA using Fugene6 transfection reagent (Roche Applied Science). The human 1061 nt Dnmt1 promoter (−967 to + 91, relative to the start ATG codon) was cloned into the Renilla luciferase reporter pLightSwitch_Prom vector (SwitchGear Genomics) and transfected with the siNupr1 or the control siRNA. Co-transfection of the pGL3-Promoter vector (Promega) was used to normalize for transfection efficiency. Medium was replaced 24 h later with fresh DMEM and incubated for an additional 48-h period. All transfections were performed at least two times, in triplicate.

### Immunoblotting

Protein extraction was performed on ice using total protein extraction buffer: 50 mM HEPES pH 7.5, 150 mM NaCl, 20% SDS, 1 mM EDTA, 1 mM EGTA, 10% glycerol, 1% Triton, 25 mM NaF, 10 μM ZnCl_2_, 50 mM DTT. Before lysis, protease inhibitor cocktail at 1:200 (Sigma-Aldrich, NUPR1340), 500 μM PMSF, 1 mM sodium orthovanadate, and 1 mM β glycerophosphate were added. Protein concentration was measured using a BCA Protein Assay Kit (Pierce Biotechnology). Protein samples (80 μg) were denatured at 95 °C and subsequently separated by SDS-PAGE gel electrophoresis. After transfer to nitrocellulose, membrane was blocking with 1% BSA, samples were probed with primary antibody followed by a horseradish peroxidase couple secondary antibody. Image acquisition was made in Fusion FX image acquisition system (Vilber Lourmat) and bands were quantified ImageJ software (NIH).

### Lung adenoma

Activation of the Kras^G12D^ oncogene in lung was induced by elimination of the conditional stop achieved by intra-tracheal inoculation of adenoviral particles (2.5 × 10^7^ pfu) expressing the Cre recombinase as previously reported^40^. Briefly, we delivered the Ad-Cre replication-deficient adenoviruses to the lungs of anesthetized mice using intratracheal intubation in a final volume of 75 μl per mouse. Mice were sacrificed after 12 weeks and lungs processes for H&E staining and SA-βGal activity. Recombination was confirmed by PCR to amplify Lox and LSL *Kras*^*G12D*^.

### Statistics

Statistical analyses were performed using the unpaired 2-tailed Student t test and Mann-Whitney test for no normally distribution, unpaired data. All test of significance were two-tailed and the level of significance was set at 0.05. Values are expressed as mean ± SD. RT-qPCR data are representative of at least 3 independent experiments with technical duplicates completed.

## Additional Information

**How to cite this article**: Grasso, D. *et al.* Pivotal Role of the Chromatin Protein Nupr1 in Kras-Induced Senescence and Transformation. *Sci. Rep.* **5**, 17549; doi: 10.1038/srep17549 (2015).

## Supplementary Material

Supplementary Information
