# Supplementary material for: Pivotal Role of the Chromatin Protein Nupr1 in Kras-Induced Senescence and Transformation
Source: Sci Rep. 2015 Nov 30;5:17549. doi: 10.1038/srep17549 (PMC4663475; doi:10.1038/srep17549)
Supplement: Supplementary Information [file srep17549-s1.pdf]

Pivotal Role of the Chromatin Protein Nupr1 in Kras-Induced Senescence and Transformation

Daniel Grasso, Jennifer Bintz, Gwen Lomberk, Maria Ines Molejon, Celine Loncle, Maria Noé Garcia, Maria Belen Lopez, Raul Urrutia, Juan L Iovanna

Supplementary information file

Supplemental Figure 1

Nupr1 regulates *Dnmt1* promotor activity in Panc1 cells. **(A)** Cells were transfected with combinations of a Dnmt1 promoter-Luc vector with siControl or siNupr1, and pSV40-RL as an internal control. After 48 h, luciferase activity was determined and expressed as the ratio of specific luciferase activity/internal standard. **(B)** Cells were transfected with combinations of a Dnmt1 promoter-Luc vector with increasing amounts of pNupr1-Flag contruct, and pSV40-RL as an internal control. After 24 h, luciferase activity was determined and expressed as the ratio of specific luciferase activity/internal standard. Means ± SD; *p<0.05, **p<0.01.

Supplemental Figure 2

Methylome analysis of siNupr1-treated MiaPaCa2 cells. Nupr1 depletion induces demethylation of 2026 genes and 887 newly methylated genes (left plot). Right plots show the number of genes related to the principals' clusters in methylated and demethylated genes.

Supplemental Figure 3

RNA from MiaPaCa-2 cells treated with siControl and siNupr1 was prepared and expression of GLTSCR2, JAG2, HABP4, CREB5, DLX3, FAT4, CPSF1, TPM4, TOB1 and RNF4 mRNA was performed by RT-qPCR. Real-time quantitative PCR was performed in a Stratagene cycler using Takara reagents. Means ± SD; *p<0.05, **p<0.01. ns = not significant.

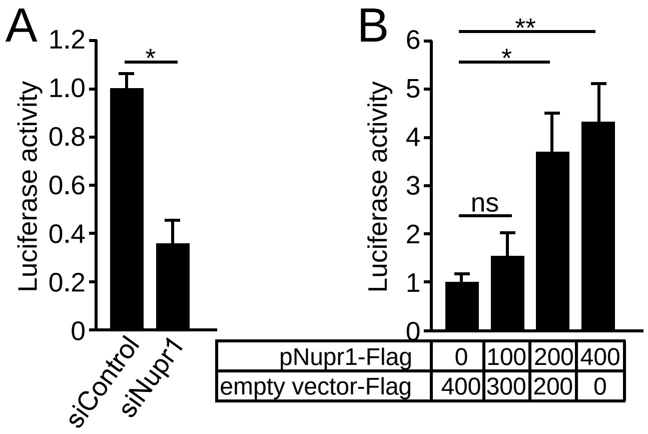
A
Luciferase activity
1.2
1.0
0.8
0.6
0.4
0.2
0
*
siControl
siNupr1
B
Luciferase activity
6
5
4
3
2
1
0
**
*
ns
pNupr1-Flag
0
100
200
400
empty vector-Flag
400
300
200
0

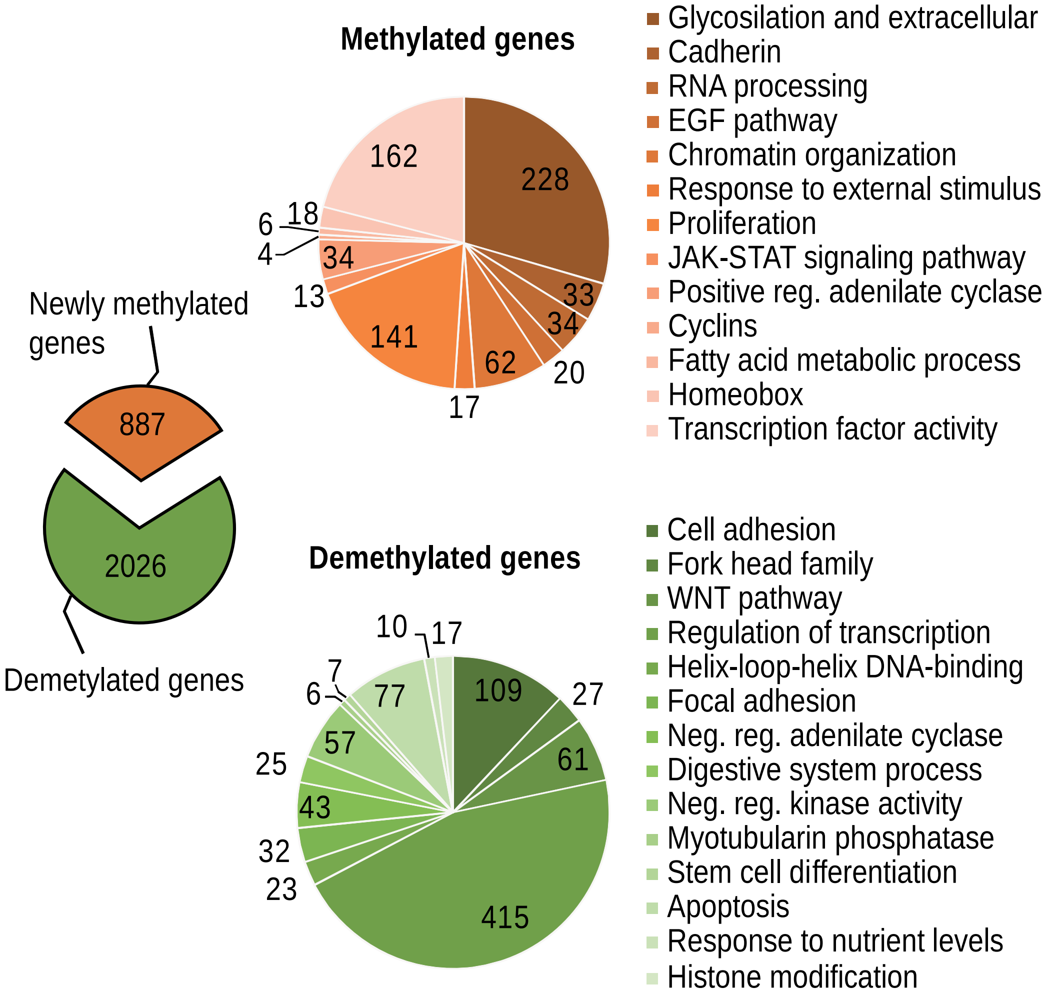
Methylated genes
228
33
34
20
62
17
141
13
34
4
6
18
162
Glycosilation and extracellular
Cadherin
RNA processing
EGF pathway
Chromatin organization
Response to external stimulus
Proliferation
JAK-STAT signaling pathway
Positive reg. adenilate cyclase
Cyclins
Fatty acid metabolic process
Homeobox
Transcription factor activity
Newly methylated genes
887
2026
Demetylated genes
Demethylated genes
109
27
61
415
23
32
43
25
57
6
7
77
10
17
Cell adhesion
Fork head family
WNT pathway
Regulation of transcription
Helix-loop-helix DNA-binding
Focal adhesion
Neg. reg. adenilate cyclase
Digestive system process
Neg. reg. kinase activity
Myotubularin phosphatase
Stem cell differentiation
Apoptosis
Response to nutrient levels
Histone modification

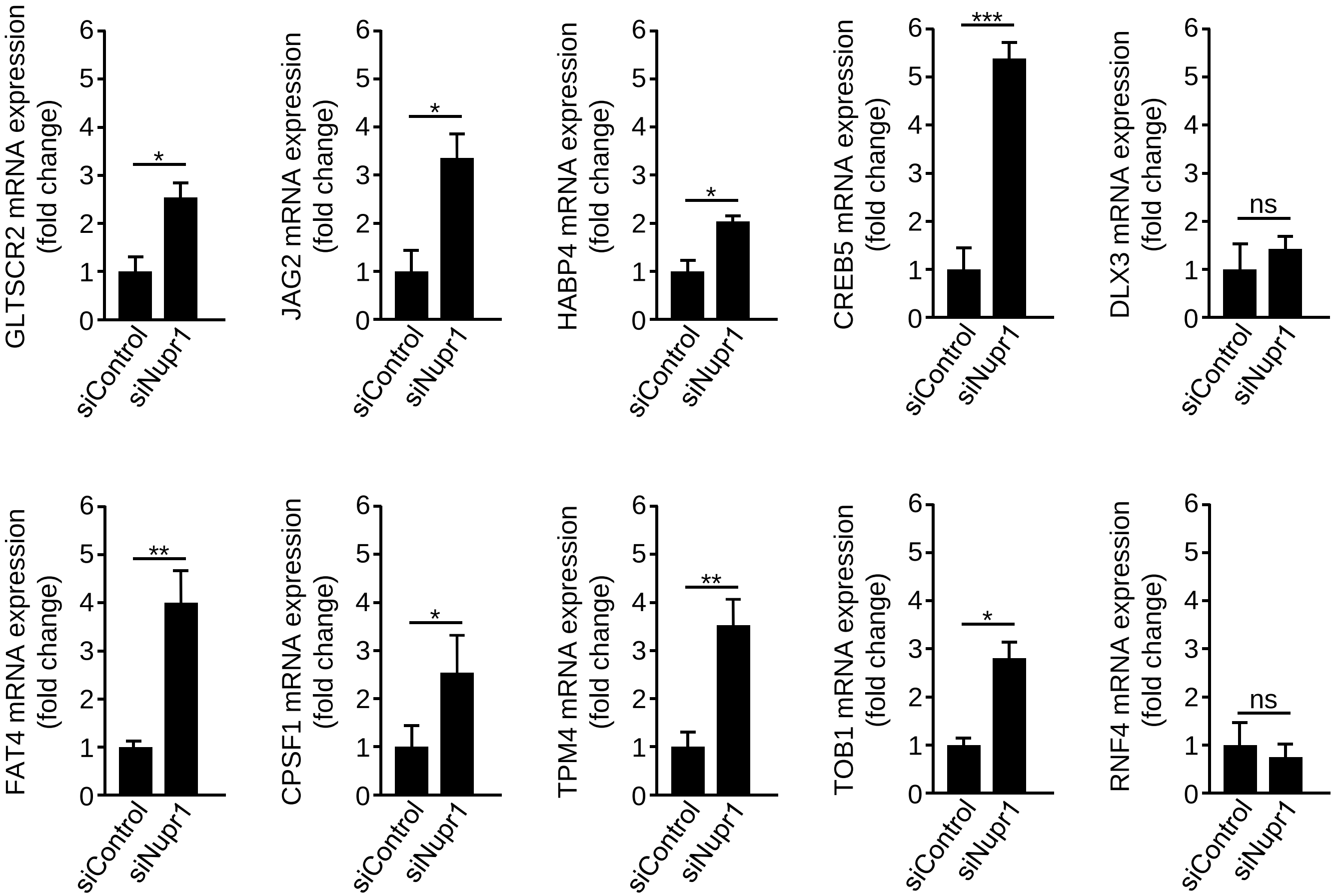
GLTSCR2 mRNA expression (fold change)
*
JAG2 mRNA expression (fold change)
*
HABP4 mRNA expression (fold change)
*
CREB5 mRNA expression (fold change)
***
DLX3 mRNA expression (fold change)
ns
FAT4 mRNA expression (fold change)
**
CPSF1 mRNA expression (fold change)
*
TPM4 mRNA expression (fold change)
**
TOB1 mRNA expression (fold change)
*
RNF4 mRNA expression (fold change)
ns
0
1
2
3
4
5
6
siControl
siNupr1

Clustered demethylated genes in siNupr1-treated miaPaca2 cells.

Annotation Cluster:	CELL ADHESION				
**Category**	**Term**	**Count**	**p Value**	**Genes**	**Fold Enrichment**
GOTERM_BP_FAT	GO:0007155~cell adhesion	93	7.77E-06	PCDHA7, AEBP1, MAEA, MYBPC2, CLDN4, PCDHA4, BCAR1, CASK, LMO7, EDIL3, CD151, CXCL12, CHAD, CTNNB1, NRCAM, DAB1, SRPX, CNTNAP1, IZUMO1, DSCAM, CYR61, FGF4, EGFR, PCDHB9, PCDHB8, MAGI1, PCDHB5, PCDHB6, EFNB1, PTPRT, CDHR5, AJAP1, SSPO, THY1, PCDHGB4, RND3, AMIGO3, HAS1, CNTN2, ROR2, ADAM12, PARVB, ITGA2B, EPDR1, PTK7, NINJ2, PCDHGC5, ITGB2, PCDHGC3, CDH3, CD72, SCRIB, PCDHAC1, ISLR, PVRL4, SORBS3, LGALS3BP, LAMB2, COL7A1, FAT4, PCDHB16, MSLN, PSTPIP1, ACAN, PKD1, PCDHA13, TECTA, FLRT2, FLRT1, HAPLN3, TNXB, MYF5, HSPG2, LGALS7, EFS, NID1, NID2, MCAM, PRPH2, TPBG, COL5A1, CDH13, LAMA4, CDH15, CDH16, NLGN4Y, HSPB11, PKP1, LAMA5, ITGA8, NLGN4X, NPHS1, ABL1, NTM, CDH11, RND3, AMIGO3, HAS1, CNTN2, ROR2, ADAM12, PARVB, ITGA2B, EPDR1, PTK7, NINJ2, PCDHGC5, ITGA8, DPP4,	1.58
GOTERM_BP_FAT	GO:0022610~biological adhesion	93	8.44E-06		1.58
SP_PIR_KEYWORDS	cell adhesion	61	9.70E-06		1.80
GOTERM_BP_FAT	GO:0016337~cell-cell adhesion	37	0.005		1.60

Annotation Cluster:	FORK HEAD FAMILY				
**Category**	**Term**	**Count**	**p Value**	**Genes**	**Fold Enrichment**
INTERPRO	IPR018122:Transcription factor, fork head, conserved site	13	3.96E-04	FOXL1, FOXJ1, FOXO3, FOXP4, FOXP1, FOXS1, FOXH1, FOXD4L2, FOXG1, FOXB2, FOXB1, FOXD4, FOXD3, PLEK2, HSFY2, E2F8, PAX2, ARID2, FEV, CUL3, FLI1, PAX7, EIF3K, EWSR1, RFX8, HSFX1, IRF4,	3.29
INTERPRO	IPR001766:Transcription factor, fork head	13	3.96E-04		3.29
UP_SEQ_FEATURE	DNA-binding region:Fork-head	13	4.04E-04		3.29
SMART	SM00339:FH	13	9.84E-04		2.96
INTERPRO	IPR011991:Winged helix repressor DNA-binding	27	0.006		1.75

Annotation Cluster:	WNT PATHWAY				
**Category**	**Term**	**Count**	**p Value**	**Genes**	**Fold Enrichment**
KEGG_PATHWAY	hsa04916:Melanogenesis	22	3.32E-05	PRKCA, WNT5A, TCF7, ADCY1, WNT10B, MAP2K1, ADCY6, FZD1, KIT, PRKX, XL1, FZD7, CTNNB1, LOC407835, EDNRB, PLCB3, MC1R, WNT9A, CALML5, PLCB1, WNT6, TUBB3, WNT8B, RAC3, FRAT1, PPP3CC, SOX17, NFATC1, PRKCA, CHP2, SKP1, DKK2, SFRP1, CCND2, TLE3, CPZ, DKK2, NXN, KREMEN1, SFRP4, WWOX, C1ORF187, BMP4, GLI1, ROR2, WNT5A, BMP4, WNT10B, TCF7, FZD1, WNT9A, WNT6, TCF7L1, FZD7, GLI1, CTNNB1, WNT8B, ROR2, BMP7, PRKX, IHH,	2.72
KEGG_PATHWAY	hsa04310:Wnt signaling pathway	25	0.001		2.03
SP_PIR_KEYWORDS	wnt signaling pathway	20	0.004		2.04
KEGG_PATHWAY	hsa05217:Basal cell carcinoma	12	0.004		2.67
GOTERM_BP_FAT	GO:0016055~Wnt receptor signaling pathway	19	0.029		1.70
KEGG_PATHWAY	hsa05217:Basal cell carcinoma	12	0.004		2.67
GOTERM_BP_FAT	GO:0007223~Wnt receptor signaling pathway, calcium modulating pathway	6	0.027		3.41
KEGG_PATHWAY	hsa04340:Hedgehog signaling pathway	10	0.035		2.19
PIR_SUPERFAMILY	PIRSF001784:int-1 transforming protein	5	0.046		3.57
INTERPRO	IPR018161:Secreted growth factor Wnt protein, conserved site	5	0.066		3.19
INTERPRO	IPR005816:Secreted growth factor Wnt protein	5	0.066		3.19
INTERPRO	IPR005817:Wnt superfamily	5	0.066		3.19
SMART	SM00097:WNT1	5	0.089		2.88

Annotation Cluster:	REGULATION OF TRANSCRIPTION				
**Category**	**Term**	**Count**	**p Value**	**Genes**	**Fold Enrichment**
GOTERM_MF_FAT	GO:0003700~transcription factor activity	115	2.01E-04	MYOD1, STAT5A, HOXD12, FOXO3, CITED1, CTNNB1, TAF5L, FLI1, ZNF445, OLIG2, PITX2, SIX5, VAX1, MECOM, PROX1, SIX6, HOXD9, ASCL2, MTF1, HOXD3, FOXG1, EDF1, NFE2L1, SOX1, ANKRD30A, TFEB, MYEF2, SRF, FOXH1, VDR, LHX1, HOXA7, ARNTL2, RUNX1, EWSR1, NKX2-2, SMAD9, GSX2, NR4A3, SMAD1, NEUROG3, MAFK, GAS7, FOXP4, VAV1, ZSCAN2, FOXP1, DLX3, HOXB1, RNF4, SALL1, MLX, DLX5, MZF1, KLF2, AEBP1, LZTS1, EVX1, HSFY2, EVX2, NR6A1, E2F8, ZNF154, FEV, GLI1, FOXS1, PAX7, POU4F3, SOX18, FOXB2, E4F1, FOXB1, RBPJL, BRD8, SNAPC2, FOXJ1, EGR4, OTX1, OTX2, ESR1, DMRT1, DMRTB1, MSC, FOXD4L2, ZSCAN18, GLIS3, IRX6, HCFC1, NFIX, ZBTB16, TCF7L1, MSX2, TSC22D2, HINFP, BCL3, BCL6, SIM1, FOXD4, FOXD3, NFATC1, SIM2, MYF6, FOXL1, HSFX1, TRIM29, TRIM28, MYF5, RCAN1, TBX1, CREB5, ISL1, VSX1, PKNOX2, IRF4, FSTL3, ZNF251, ZFP92, EPC1, TWIST1, YY2, ZNF507, TADA2B, ZNF648, C14ORF43, TOX2, PRDM8, BAZ1A, PTRF, HES4, ZNF785, ZNF382, CRTC2, ZNF517, CRTC1, FIGLA, ZNF74, TCF7, RYBP, KLF14, ZNF526, ZNF629, NOTCH3, TCEB3C, TCEB3B, TAF1C, ZNF534, ZFP41, ZNF296, ZNF295, RLIM, ZNF677, FERD3L, ZNF354C, GMEB2, TLE3, ZNF143, ZNF689, CAND1, ZNF746, NSD1, ZNF276, PTOV1, ZNF275, ZFP64, ZNF467, ZNF558, ZNF324B, ZNF366, MLLT1, BAZ2A, JARID2, ZNF770, ZNF667, ZNF669, ZNF664, PHF19, ZNF461, ZNF572, ZNF768, RBM4, TCEAL5, CBX2, OLIG3, MIER2, MYOCD, MIER3, TFB2M, ZNF576, MLL2, ARID1B, GRHL2, SLTM, ZNF433, SETD1A, KEAP1, CXXC1, OVOL1, SNF8, ZNF425, FEZF1, EBF2, ZNF418, HABP4, ZNF319, PAX2, PCGF3,PAX7, SOX18, SOX17, ZNF721, BANP, NPAS4, COBRA1, EYA4, FAM120B, ZNF710, ING5, GLIS1, TRIB3, CTCFL, PRDM16, ARID2, HIC2, PRDM15, NPAS3, BAHD1, KAT2A, PPP1R13L, SETD7, FSTL3, ZNF629, POLRMT, POLR2L, POLR3E, HLTF, CD3EAP, BCL6B, DNAJB6, PTGES2, PSMD9, JMY, PRPF6, DTX1, TOB1, C10ORF2, YBX2, HSF2BP, PHRF1, RRAGC, SIRT7, TSNAX, STRA13, FBXO18, AKAP8, HIST1H4K, PEG10, TXNDC12, GLI4, PCBP3, TOP3A, THAP4, MAP1S, ZNF646, LIG1, PNKP, MYO18A, SPHK1, CCDC79, FBXL19, ABTB2, PMS1, EGFR, LIN28A, RFX, ABL1, XP1, TRAK2, TRAK1, ZNF646, XLIF, PFN1, XBMP4, BMP3, MAP2K1, BMP7, CCDC79, IHH, JMY, SIGIRR, CARD11, SORBS3, FADS1, FZD1, SOD2, CASK, ABL1, TNFRSF4, BMP4? SNRNP70, CUL3, INTS6, FAM53A, STAG3, CCNA1, WWOX, CIZ1, LIG1, KIAA0649, PNKP, FBXL3, PNMA1, TP53TG3, ADAP1, PPAN, UBASH3B, C19ORF22, RECQL5, DUSP23, LGALS7, CAMK2N2, SYNE2, PKP1, ATP2A3, LTC4S, SENP6, RRP1B, NUBP2, FANCA, CCNF, NOC2L, ANKRD23, STH, NAIF1, SSU72, NXT1, NHLRC1, QTRT1, MLF2, TRIM11, SF3B2, GCH1, SRRT, STK40, BUB3, EIF4ENIF1, CAPN7, BRD3, PPAN-P2RY11, AFF2, CRIPAK, STRADB, SF3A2, PLEKHF1, LRP1, FYTTD1, MEX3C, ZNF259, OGFR, TP53INP2, MAEA, SNRPD1, FGF12, STK39, DND1, IMP4, PMS1, MTUS1, NET1, FAM82A2, DYX1C1, NEIL1, TATDN2, PNPLA7, PSMA1, MAD2L1BP, BMP2K, DDX31, CAV2, DSCR6, STK11, SSH3, SIPA1, X, MAPKAPK3, TCL1A, ANAPC11, ZNF330, HNRNPM, FBXW7, PTK6, EIF3K, STK19, EPB41, CINP, KCTD5, CDKN1A, NXN, KIF23, AKT1, OSR2, EPN3, NOL4, DYNLT3, MID1IP1, RFWD2, SBF1, CHFR, CPSF1, KPNA1, GLTSCR2, BRSK1, ADPRHL2, RGS12, GCHFR, PARK2, CBS,	1.39
SP_PIR_KEYWORDS	transcription regulation	204	3.85E-04		1.26
SP_PIR_KEYWORDS	Transcription	207	4.90E-04		1.25
GOTERM_MF_FAT	GO:0030528~transcription regulator activity	163	7.26E-04		1.27
GOTERM_BP_FAT	GO:0006350~transcription	214	0.001		1.21
GOTERM_MF_FAT	GO:0043565~sequence-specific DNA binding	73	0.002		1.42
SP_PIR_KEYWORDS	dna-binding	184	0.002		1.23
GOTERM_MF_FAT	GO:0003677~DNA binding	232	0.004		1.17
GOTERM_BP_FAT	GO:0045449~regulation of transcription	252	0.006		1.15
GOTERM_BP_FAT	GO:0006355~regulation of transcription, DNA-dependent	177	0.008		1.19
GOTERM_BP_FAT	GO:0051252~regulation of RNA metabolic process	178	0.014		1.17
SP_PIR_KEYWORDS	nucleus	363	0.126		1.06

Annotation Cluster:	HELIX-LOOP-HELIX DNA-BINDING				
**Category**	**Term**	**Count**	**p Value**	**Genes**	**Fold Enrichment**
UP_SEQ_FEATURE	domain:Helix-loop-helix motif	19	0.004	MYF6, MYOD1, MYF5, TFEB, FIGLA, NPAS4, NEUROG3, MSC, FERD3L, ASCL2, NPAS3, OLIG3, HES4, MLX, OLIG2, ARNTL2, SIM1, SIM2, TWIST1, EBF2, CREB5, MAFK, NFE2L1,	2.06
INTERPRO	IPR001092:Basic helix-loop-helix dimerisation region bHLH	19	0.005		2.02
SMART	SM00353:HLH	19	0.015		1.82

INTERPRO	IPR011598:Helix-loop-helix DNA-binding	14	0.019		2.02
UP_SEQ_FEATURE	DNA-binding region:Basic motif	21	0.035		1.62

Annotation Cluster:	FOCAL ADHESION				
**Category**	**Term**	**Count**	**p Value**	**Genes**	**Fold Enrichment**
GOTERM_CC_FAT	GO:0005912~adherens junction	24	0.003	CAV2, ENAH, BCAR1, ASZ1, LMO7, KEAP1, EVL, AJAP1, CDH3, RHOU, APBB1IP, MLF2, SCRIB, CTNNB1, PVRL4, TNS3, SORBS3, LAYN, LASP1, EPB41L5, DSP, PAK1, PARVB, ITGA2B, PKP1, SHROOM4, CASK, AQP1, STK39, EGFR, SLC26A7, SLC16A8,	1.91
GOTERM_CC_FAT	GO:0070161~anchoring junction	25	0.006		1.79
GOTERM_CC_FAT	GO:0005925~focal adhesion	16	0.017		1.93
GOTERM_CC_FAT	GO:0005924~cell-substrate adherens junction	16	0.024		1.86
GOTERM_CC_FAT	GO:0030055~cell-substrate junction	16	0.037		1.76
GOTERM_CC_FAT	GO:0016323~basolateral plasma membrane	25	0.039		1.52

Annotation Cluster:	NEGATIVE REGULATION OF ADENILATE CYCLASE ACTIVITY				
**Category**	**Term**	**Count**	**p Value**	**Genes**	**Fold Enrichment**
GOTERM_BP_FAT	GO:0030814~regulation of cAMP metabolic process	18	0.005	GNAZ, MCHR1, ADCY1, OPRL1, NF1, ADCY6, NPR3, TIMP2, CRHR1, EDNRB, SSTR5, GRM3, ADRB1, CHRM4, NTRK1, GRM7, NTRK2, GLP1R, HIST1H4K, GNA11, KISS1R, MC1R, HTR5A, TUBB3, EGFR, RXFP3, SPHK1, RCAN1, PCLO, GRM1, MCTP1, SSTR4, CRHR2, SSTR3, NPY, HTR6, TBXA2R, ITGA8, CACNA2D3, CACNG1, TPM4, PRKX, ITGA2B,	2.08
GOTERM_BP_FAT	GO:0045761~regulation of adenylate cyclase activity	17	0.006		2.11
GOTERM_BP_FAT	GO:0031279~regulation of cyclase activity	17	0.008		2.05
GOTERM_BP_FAT	GO:0030817~regulation of cAMP biosynthetic process	17	0.010		2.01
GOTERM_BP_FAT	GO:0051339~regulation of lyase activity	17	0.010		2.01
GOTERM_BP_FAT	GO:0030799~regulation of cyclic nucleotide metabolic process	18	0.013		1.90
GOTERM_BP_FAT	GO:0007194~negative regulation of adenylate cyclase activity	11	0.015		2.38
GOTERM_BP_FAT	GO:0031280~negative regulation of cyclase activity	11	0.015		2.38
GOTERM_BP_FAT	GO:0051350~negative regulation of lyase activity	11	0.015		2.38
GOTERM_BP_FAT	GO:0006140~regulation of nucleotide metabolic process	18	0.016		1.85
GOTERM_BP_FAT	GO:0030808~regulation of nucleotide biosynthetic process	17	0.021		1.84
GOTERM_BP_FAT	GO:0030802~regulation of cyclic nucleotide biosynthetic process	17	0.021		1.84
GOTERM_BP_FAT	GO:0019932~second-messenger-mediated signaling	30	0.023		1.52
GOTERM_BP_FAT	GO:0019935~cyclic-nucleotide-mediated signaling	19	0.024		1.74
GOTERM_BP_FAT	GO:0007193~inhibition of adenylate cyclase activity by G-protein signaling	8	0.046		2.38
GOTERM_BP_FAT	GO:0019933~cAMP-mediated signaling	14	0.053		1.76
GOTERM_BP_FAT	GO:0007187~G-protein signaling, coupled to cyclic nucleotide second messenger	16	0.057		1.66
GOTERM_BP_FAT	GO:0007188~G-protein signaling, coupled to cAMP nucleotide second messenger	11	0.173		1.54
GOTERM_BP_FAT	GO:0045762~positive regulation of adenylate cyclase activity	8	0.186		1.70
GOTERM_BP_FAT	GO:0031281~positive regulation of cyclase activity	8	0.198		1.67
GOTERM_BP_FAT	GO:0051349~positive regulation of lyase activity	8	0.223		1.62
GOTERM_BP_FAT	GO:0007190~activation of adenylate cyclase activity	7	0.313		1.52
KEGG_PATHWAY	hsa05414:Dilated cardiomyopathy	9	0.478		1.20
GOTERM_BP_FAT	GO:0007189~activation of adenylate cyclase activity by G-protein signaling pathw	3	0.860		0.89
GOTERM_BP_FAT	GO:0010578~regulation of adenylate cyclase activity involved in G-protein signali	3	0.860		0.89
GOTERM_BP_FAT	GO:0010579~positive regulation of adenylate cyclase activity by G-protein signali	3	0.860		0.89

Annotation Cluster:	DIGESTIVE SYSTEM PROCESS				
**Category**	**Term**	**Count**	**p Value**	**Genes**	**Fold Enrichment**
GOTERM_BP_FAT	GO:0022600~digestive system process	9	0.006	KCNMA1, VDR, KCNN4, SCT, LDLR, SLC26A7, STATH, IREB2, MUC6, SLC22A2, UGT1A6, UGT1A8, NPY, DPP3, LAP3, F12, AEBP1, PEPD, TRHDE, DPEP3, PGCP, CPA1, DPP6, DPP4, CPZ,	3.16
GOTERM_BP_FAT	GO:0046541~saliva secretion	3	0.038		8.94
GOTERM_BP_FAT	GO:0007589~body fluid secretion	5	0.186		2.21
GOTERM_BP_FAT	GO:0007586~digestion	11	0.231		1.44
GOTERM_MF_FAT	GO:0008238~exopeptidase activity	12	0.059		1.84
GOTERM_MF_FAT	GO:0004177~aminopeptidase activity	6	0.106		2.36
SP_PIR_KEYWORDS	Aminopeptidase	4	0.347		1.92
GOTERM_MF_FAT	GO:0008235~metalloexopeptidase activity	7	0.098		2.17

GOTERM_MF_FAT	GO:0004181~metallocarboxypeptidase activity	5	0.157		2.36
GOTERM_MF_FAT	GO:0004180~carboxypeptidase activity	5	0.385		1.59
INTERPRO	IPR000834:Peptidase M14, carboxypeptidase A	3	0.576		1.58
SMART	SM00631:Zn_pept	3	0.634		1.43
SP_PIR_KEYWORDS	carboxypeptidase	3	0.739		1.17

Annotation Cluster:	NEGATIVE REGULATION OF KINASE ACTIVITY				
Category	Term	Count	p Value	Genes	Fold Enrichment
GOTERM_BP_FAT	GO:0043086~negative regulation of catalytic activity	34	0.025	GNAZ, MCHR1, ADCY1, ADCY6, PRKAG2, TRIB3, ANAPC11, AKT1, EDNRB, PCSK9, MLLT1, GCHFR, BUB3, PSMD9, PRKCA, ANAPC5, OPRL1, NF1, NEIL1, CRIPAK, NPR3, THY1, SH3BP5, PSMA1, SSTR5, GRM3, CDKN1A, CHRM4, GRM7, TSC2, ZFYVE28, CAND1, DNAJB6, DUSP6, CCK, MAP4K1, KIT, TNFRSF4, S1PR2, KISS1R, DAB1, DGKE, DIRAS3, PAK1, FRS2, EGFR, MAP2K1, SPHK1, STRADB, GRM1, CARD10, LRP1, CCND2, CD81, MAPK8IP3, CBS, VLDLR,	1.46
GOTERM_BP_FAT	GO:0006469~negative regulation of protein kinase activity	13	0.059		1.78
GOTERM_BP_FAT	GO:0045859~regulation of protein kinase activity	38	0.071		1.31
GOTERM_BP_FAT	GO:0033673~negative regulation of kinase activity	13	0.072		1.72
GOTERM_BP_FAT	GO:0051348~negative regulation of transferase activity	13	0.105		1.61

Annotation Cluster:	MYOTUBULARIN PHOSPHATASE				
Category	Term	Count	p Value	Genes	Fold Enrichment
INTERPRO	IPR004182:GRAM	5	0.066	MTMR1, SBF1, SBF2, TBC1D9, WBP2, MTMR9,	3.19
SMART	SM00568:GRAM	5	0.089		2.88
UP_SEQ_FEATURE	domain:Myotubularin phosphatase	4	0.098		3.54
INTERPRO	IPR010569:Myotubularin-related	4	0.102		3.47
INTERPRO	IPR017906:Myotubularin phosphatase	4	0.102		3.47
UP_SEQ_FEATURE	domain:GRAM	4	0.115		3.30

Annotation Cluster:	STEM CELL DIFFERENTIATION				
Category	Term	Count	p Value	Genes	Fold Enrichment
GOTERM_BP_FAT	GO:0048863~stem cell differentiation	7	0.047	LIF, PAX7, TCL1A, KIT, LIN28A, TCF7L1, FGF4,	2.61
GOTERM_BP_FAT	GO:0019827~stem cell maintenance	5	0.153		2.38
GOTERM_BP_FAT	GO:0048864~stem cell development	5	0.169		2.29

Annotation Cluster:	APOPTOSIS				
Category	Term	Count	p Value	Genes	Fold Enrichment
GOTERM_BP_FAT	GO:0010942~positive regulation of cell death	46	0.082	CCK, MMP9, ZBTB16, FOXO3, SCRIB, MCF2L, GCH1, MSX2, CUL3, AKT1, VDR, BOK, TNFRSF19, PCSK9, PPP3CC, BCL3, BCL6, MX1, CD5, WWOX, NET1, KCNMA1, BMP4, PRKCA, ARHGEF4, OBSCN, ARHGEF2, ARHGEF18, NF1, CIDEA, GAL, VAV1, DAPK1, JMY, PLEKHF1, TXNDC12, CDKN1A, UNC13D, SSTR3, RASGRF2, RASGRF1, NAIF1, BIK, ABL1, BMP7, DAP3, MAEA, SNCB, STAT5A, BCAR1, JAG2, PAX2, EDNRB, , PAX7, DDAH2, FGF4, IHH, NMNAT3, EGFR, ESR1, CARD10, CARD11, NME5, TNFAIP8, MYO18A, KIT, TNFRSF4, CARD9, SPHK1, PDE3A, STRADB, SOD2, CDH13, SFRP1, NTRK1, DNAJB6,	1.26
GOTERM_BP_FAT	GO:0043065~positive regulation of apoptosis	45	0.093		1.25
GOTERM_BP_FAT	GO:0043068~positive regulation of programmed cell death	45	0.105		1.24
GOTERM_BP_FAT	GO:0006917~induction of apoptosis	33	0.169		1.23
GOTERM_BP_FAT	GO:0012502~induction of programmed cell death	33	0.173		1.23
GOTERM_BP_FAT	GO:0010941~regulation of cell death	76	0.203		1.11
GOTERM_BP_FAT	GO:0043067~regulation of programmed cell death	75	0.235		1.10
GOTERM_BP_FAT	GO:0042981~regulation of apoptosis	73	0.289		1.08
GOTERM_BP_FAT	GO:0043069~negative regulation of programmed cell death	29	0.685		0.96
GOTERM_BP_FAT	GO:0060548~negative regulation of cell death	29	0.690		0.96
GOTERM_BP_FAT	GO:0043066~negative regulation of apoptosis	27	0.789		0.91
GOTERM_BP_FAT	GO:0006916~anti-apoptosis	14	0.890		0.81

Annotation Cluster:	RESPONSE TO NUTRIENT LEVELS				
Category	Term	Count	p Value	Genes	Fold Enrichment
GOTERM_BP_FAT	GO:0031668~cellular response to extracellular stimulus	8	0.288	VDR, CYP24A1, CDKN1A, MYOCD, MAP1LC3A, FADS1, PCSK9, CTSD, UGT1A6, UGT1A8,	1.49
GOTERM_BP_FAT	GO:0031669~cellular response to nutrient levels	6	0.308		1.63
GOTERM_BP_FAT	GO:0042594~response to starvation	5	0.473		1.42
GOTERM_BP_FAT	GO:0009267~cellular response to starvation	4	0.510		1.49

Annotation Cluster:	HISTONE MODIFICATION				
Category	Term	Count	p Value	Genes	Fold Enrichment
GOTERM_BP_FAT	GO:0043543~protein amino acid acylation	8	0.235	ING5, NMT2, KAT2A, EPC1, TAF5L, IRF4, MAP6D1, BRD8, PRMT8, RBM4, CTCFL, NSD1, BAZ2A, POLR2L, INTS6, EDF1, ZNF768,	1.59

GOTERM_BP_FAT	GO:0043966~histone H3 acetylation	4	0.374		1.83
GOTERM_BP_FAT	GO:0016573~histone acetylation	6	0.376		1.49
GOTERM_BP_FAT	GO:0006473~protein amino acid acetylation	6	0.444		1.38
GOTERM_BP_FAT	GO:0043967~histone H4 acetylation	3	0.509		1.79
GOTERM_BP_FAT	GO:0016570~histone modification	11	0.577		1.07
GOTERM_CC_FAT	GO:0000123~histone acetyltransferase complex	5	0.586		1.23
GOTERM_BP_FAT	GO:0016569~covalent chromatin modification	11	0.619		1.04
GOTERM_MF_FAT	GO:0004468~lysine N-acetyltransferase activity	4	0.636		1.24
GOTERM_MF_FAT	GO:0004402~histone acetyltransferase activity	4	0.636		1.24
GOTERM_CC_FAT	GO:0016591~DNA-directed RNA polymerase II, holoenzyme	6	0.796		0.91

Clustered methylated genes in siNupr1-treated miaPaca2 cells.

Annotation Cluster: **GLYCOSYLATION AND EXTRACELLULAR REGION**

Category	Term	Count	p Value	Genes	Fold Enrichment
UP_SEQ_FEATURE	glycosylation site:N-linked (GlcNAc...)	180	8.61E-04	KERA, PTGS2, LTBP3, PDGFA, C20ORF103, OVCH2, TLR3, POSTN, GRIN3A, TSPAN9, MMRN1, CNGB3, CDH22, ADAM2, OR6C1, GXYLT1, LPHN1, B3GALTL, PI15, CLRN3, MFI2, OR5M3, IL22, GABRR3, TNFAIP6, OR5H14, F3, ASPHD2, HTR7, OR13F1, TMPRSS11B, PTGFRN, IBSP, NAGLU, SCN1A, GAL3ST4, SLC38A2, CYSLTR1, DRD2, TMEM81, OR2T4, OR11G2, UMOD, OR2L3, OR8U1, GREM2, ITGB1, ZPLD1, KDELC1, FGG, PVRL1, BCHE, TEK, B3GNT6, SCN9A, PCDHA10, ENTPD6, FGL2, EGF, OLFM1, FAM55D, MEST, FCER1A, PTH2R, ACER1, MGAT4C, ITGA1, SLC6A15, MSTN, TM2D3, EMILIN3, SERPINI2, EPHA3, HYOU1, TMEM117, OR12D2, ATP13A1, OR6K3, WIF1, AREG, OR8D2, MTNR1A, C9ORF11, PNLIP, NXPH2, OR10A4, PCDHA9, NDST4, ARSE, OR52A1, NXPH4, ITIH5L, HEXA, PCDHA5, ANO1, OR4K17, ARSK, OR10A7, TOR3A, OR4C16, OR4C15, OR9K2, TNFRSF11A, RSPO1, OR2T10, OR14J1, TGFA, OR8G1, METTL9, GPR174, HYAL2, ADAM10, RXFP1, CNTNAP4, NRXN3, PCDHGB7, MESDC2, OXGR1, SLC7A11, LYZL1, LYZL2, ACVR2B, KIAA1199, GRM7, CTSO, OR5V1, OR4N5, OR5H6, ADAM15, CD101, OR8H3, C5, GPAA1, TSHB, PCDHB12, DCN, EDEM1, SCARF2, LINGO2, IGSF11, GPM6A, LRRTM4, TSPAN33, PKD2, OR5B12, TOR1B, SLC4A7, PKD1, CCBP2, HTR3B, LY75, OR9G9, FAM174A, SLC12A1, IL5, FLT3, B3GALT1, SI, SUN2, TSPAN14, EDA2R, OR9G1, NPY1R, COL5A2, KIAA0564, FZD6, OR5T3, CDH12, RNF43, AFP, DKK1, LIPK, EPGN, CDH17, CDH18, CDH19, KREMEN1, GPR113, SUMF1, OR10Q1, CD302, OR6X1, CD40LG, ODAM, REG1A, CSN3, AADACL2, X, HTRA1, XORF53, SLITRK1, RBP4, ADCYAP1, SPP2, CXCL13, INSL5, PRDX4, CCL3L3, TGFA, FAM24B, FAM24A, AMBN, MAGT1, C1QL3, VOPP1, SMR3B, CXCL1, MMGT1, TAC1, C1QTNF7, PRRG4, NMU, IL8, BMPR1B, C4ORF7, RCN2, IL18, GPI, ZFP91, CNTF, CMTM7, HDLBP, VASH1, SCGN, KRTAP1-1, X, KRTAP1-3, HDLBPX,	1.24
SP_PIR_KEYWORDS	glycoprotein	184	0.002		1.22
SP_PIR_KEYWORDS	signal	139	0.007		1.22
UP_SEQ_FEATURE	signal peptide	139	0.008		1.22
GOTERM_MF_FAT	GO:0005125~cytokine activity	15	0.011		2.12
SP_PIR_KEYWORDS	cytokine	13	0.025		2.06
GOTERM_CC_FAT	GO:0005615~extracellular space	35	0.036		1.41
KEGG_PATHWAY	hsa04060:Cytokine-cytokine receptor interaction	16	0.059		1.64
SP_PIR_KEYWORDS	Secreted	71	0.072		1.20
GOTERM_CC_FAT	GO:0005576~extracellular region	83	0.123		1.14
GOTERM_CC_FAT	GO:0044421~extracellular region part	42	0.146		1.21

Annotation Cluster: **CADHERIN**

Category	Term	Count	p Value	Genes	Fold Enrichment
GOTERM_BP_FAT	GO:0007156~homophilic cell adhesion	13	0.003	PCDHA9, PCDHA5, PCDHGB7, PCDHB12, ITGB1, CDH22, CDH12, PVRL1, CDH17, CDH18, CDH19, PKD1, PCDHA10, CLDN18, SCARF2, BARX2, CD40LG, TEK, BMPR1B, IBSP, CNTNAP4, NRXN3, ITGA1, POSTN, IGSF11, ARVCF, TNFAIP6, ADAM2, PPFIA1, MMRN1, PKD2, AMBN, ADAM15,	2.77
UP_SEQ_FEATURE	domain:Cadherin 5	10	0.006		2.99
UP_SEQ_FEATURE	domain:Cadherin 3	10	0.010		2.79
UP_SEQ_FEATURE	domain:Cadherin 4	10	0.010		2.79
GOTERM_BP_FAT	GO:0016337~cell-cell adhesion	19	0.010		1.92
SMART	SM00112:CA	10	0.011		2.72
UP_SEQ_FEATURE	domain:Cadherin 1	10	0.012		2.71
UP_SEQ_FEATURE	domain:Cadherin 2	10	0.012		2.71
INTERPRO	IPR002126:Cadherin	10	0.013		2.65
INTERPRO	IPR000233:Cadherin cytoplasmic region	4	0.027		6.04
PIR_SUPERFAMILY	PIRSF002504:cadherin	4	0.040		5.14
SP_PIR_KEYWORDS	cell adhesion	22	0.066		1.49
GOTERM_BP_FAT	GO:0022610~biological adhesion	33	0.090		1.32
GOTERM_BP_FAT	GO:0007155~cell adhesion	33	0.092		1.32
UP_SEQ_FEATURE	domain:Cadherin 6	6	0.113		2.34
INTERPRO	IPR013164:Cadherin, N-terminal	5	0.143		2.47

Annotation Cluster: **RNA PROCESSING**

Category	Term	Count	p Value	Genes	Fold Enrichment
GOTERM_BP_FAT	GO:0042254~ribosome biogenesis	12	0.004	DIS3, DCAF13, RNASEL, RPS19, DKC1, TSR2, UTP6, NOP58, GNL3L, HEATR1, RSL24D1, NOP10, FARS2, LIN28B, INTS6, TRNT1, UTP3, HMGB1, ZBTB33, PDLIM3, TAF7L, TSPYL1, CIR1, MYH1, SOX11, ZFX, NXF5, FOXP1, WASL, SH3D19, PPARGC1A, TARBP1, PPIH, LARP7,	2.75
GOTERM_BP_FAT	GO:0006364~rRNA processing	10	0.006		3.04
SP_PIR_KEYWORDS	ribosome biogenesis	7	0.006		4.17
GOTERM_BP_FAT	GO:0016072~rRNA metabolic process	10	0.007		2.91
GOTERM_BP_FAT	GO:0034470~ncRNA processing	14	0.017		2.09
SP_PIR_KEYWORDS	rrna processing	7	0.019		3.28
GOTERM_BP_FAT	GO:0022613~ribonucleoprotein complex biogenesis	12	0.058		1.86
GOTERM_BP_FAT	GO:0034660~ncRNA metabolic process	14	0.069		1.70
GOTERM_CC_FAT	GO:0005730~nucleolus	25	0.627		0.99
GOTERM_BP_FAT	GO:0006396~RNA processing	19	0.674		0.97

Annotation Cluster: **EGF PATHWAY**

Category	Term	Count	p Value	Genes	Fold Enrichment
INTERPRO	IPR013032:EGF-like region, conserved site	20	0.007	EPHA3, TNFRSF11A, GPR113, HYAL2, EPGN, ADAM15, ADAM2, ITGB1, PTGS2, TGFA, AREG, MMRN1, CNTNAP4, LTBP3, NRXN3, TEK, UMOD, WIF1, EGF, SCARF2	1.96
SMART	SM00181:EGF	15	0.008		2.19
INTERPRO	IPR006210:EGF-like	15	0.011		2.14
UP_SEQ_FEATURE	domain:EGF-like	8	0.017		2.99

INTERPRO	IPR000742:EGF-like, type 3	14	0.018		2.07
SP_PIR_KEYWORDS	egf-like domain	15	0.031		1.87
INTERPRO	IPR013111:EGF, extracellular	6	0.068		2.73
INTERPRO	IPR006209:EGF	9	0.076		2.03
UP_SEQ_FEATURE	domain:EGF-like 3	6	0.123		2.28
UP_SEQ_FEATURE	domain:EGF-like 1	8	0.130		1.90
UP_SEQ_FEATURE	domain:EGF-like 2	5	0.373		1.62
UP_SEQ_FEATURE	domain:EGF-like 5	3	0.425		2.08
UP_SEQ_FEATURE	domain:EGF-like 4	3	0.609		1.47

Annotation Cluster: CHROMATIN ORGANIZATION

Category	Term	Count	p Value	Genes	Fold Enrichment
INTERPRO	IPR009072:Histone-fold	8	0.003	SUPT3H, HIST1H2BC, HIST4H4, HIST1H4A, HIST1H2BL, TAF9B, HIST1H3A, HIST1H2AI, H2AFJ, HIST1H4J, HIST1H3H, HIST1H1C, HIST1H1A, CENPF, X, TSPYL1, ARID4A, BAZ1B, CHD1, UTP3, ENY2, HMGB1, KDM5B, AEBP2, HDAC10, BANP, HMG20B, RPS6KA5, SUZ12, MSL1, DKC1, RFC1, PELO, MBD2, FANCI, HIST1H3A, CD40LG,C5, KDM1B, MYH1, G3BP1, MYH4, RAP1B, RUNX1, RAB21, SUN2, TEX12, TAL1, DMC1, FGG, PSMG2, TUBAL3, WASL, SLC38A2, EPAS1, RXRA, UBE2CBP, RNF43, TAL1, SORBS2, TRIM31, FBXO43, SCN9A,	4.25
GOTERM_BP_FAT	GO:0065004~protein-DNA complex assembly	10	0.005		3.07
GOTERM_BP_FAT	GO:0006333~chromatin assembly or disassembly	12	0.006		2.64
GOTERM_CC_FAT	GO:0000786~nucleosome	8	0.007		3.51
GOTERM_BP_FAT	GO:0006325~chromatin organization	24	0.009		1.77
GOTERM_BP_FAT	GO:0006334~nucleosome assembly	9	0.010		2.99
GOTERM_BP_FAT	GO:0031497~chromatin assembly	9	0.012		2.89
GOTERM_BP_FAT	GO:0051276~chromosome organization	28	0.015		1.61
GOTERM_BP_FAT	GO:0034728~nucleosome organization	9	0.018		2.70
INTERPRO	IPR007125:Histone core	6	0.020		3.82
SP_PIR_KEYWORDS	nucleosome core	6	0.025		3.58
GOTERM_CC_FAT	GO:0000785~chromatin	14	0.029		1.94
SP_PIR_KEYWORDS	chromosomal protein	11	0.031		2.17
GOTERM_CC_FAT	GO:0032993~protein-DNA complex	8	0.035		2.57
GOTERM_BP_FAT	GO:0006323~DNA packaging	9	0.058		2.15
KEGG_PATHWAY	hsa05322:Systemic lupus erythematosus	8	0.073		2.17
PIR_SUPERFAMILY	PIRSF002048:histone H2A	3	0.113		5.14
SMART	SM00414:H2A	3	0.134		4.64
INTERPRO	IPR002119:Histone H2A	3	0.140		4.53
SP_PIR_KEYWORDS	citrullination	3	0.178		3.90
SP_PIR_KEYWORDS	chromatin regulator	11	0.211		1.48
SP_PIR_KEYWORDS	methylation	12	0.225		1.42
GOTERM_CC_FAT	GO:0005694~chromosome	21	0.227		1.26
GOTERM_CC_FAT	GO:0044427~chromosomal part	18	0.233		1.29
GOTERM_BP_FAT	GO:0016568~chromatin modification	13	0.277		1.33
GOTERM_BP_FAT	GO:0034622~cellular macromolecular complex assembly	14	0.349		1.23
UP_SEQ_FEATURE	cross-link:Glycyl lysine isopeptide (Lys-Gly) (interchain with G-Cter in ubiquitin)	9	0.399		1.29
GOTERM_BP_FAT	GO:0034621~cellular macromolecular complex subunit organization	15	0.401		1.17
SP_PIR_KEYWORDS	isopeptide bond	12	0.559		1.08
SP_PIR_KEYWORDS	ubl conjugation	20	0.667		0.97

Annotation Cluster: RESPONSE TO EXTERNAL STIMULUS

Category	Term	Count	p Value	Genes	Fold Enrichment
GOTERM_BP_FAT	GO:0032103~positive regulation of response to external stimulus	7	0.026	FCER1A, ZFP91, ADAM10, CNTF, IL8, F3, FABP4, TAC1, SH2D1A, CARD9, TLR3, PTGS2, ACVR2B, PTGS2, SI, ILDR2, EIF2B2,	3.06
GOTERM_BP_FAT	GO:0031349~positive regulation of defense response	6	0.119		2.30
GOTERM_BP_FAT	GO:0050729~positive regulation of inflammatory response	3	0.291		2.80
GOTERM_BP_FAT	GO:0050727~regulation of inflammatory response	4	0.513		1.47
GOTERM_BP_FAT	GO:0034284~response to monosaccharide stimulus	5	0.086		2.97
GOTERM_BP_FAT	GO:0009746~response to hexose stimulus	5	0.086		2.97
GOTERM_BP_FAT	GO:0009743~response to carbohydrate stimulus	5	0.166		2.33

GOTERM_BP_FAT	GO:0009749~response to glucose stimulus	3	0.481		1.86

Annotation Cluster:	PROLIFERATION				
Category	Term	Count	p Value	Genes	Fold Enrichment
GOTERM_BP_FAT	GO:0045741~positive regulation of epidermal growth factor receptor activity	3	0.012	EPGN, TGFA, EGF, FCER1A, CARD9, IL5, PTGS2, TLR3, TAC1, EDA2R, SHOC2, RICTOR, MBD2, TRAT1, ZFP91, ACVR2B, CNTF, CDKN2B, F3, MTOR, NMU, AMOT, PPARGC1A, PSMG2, CENPF, ANAPC10, CEP76, DAZL, PTPLAD1, C5, ITGA1, GPI, IL8, EPAS1, PDGFA, IL18, NAA15, CCDC88C, NF1, DGKH, IL22, CCNE2, FABP4, PRKACB, SOCS2, DRD2, PRDX4, TANK, RPS6KA5, PKD2, PKD1, LRRK2, ITGB1, ADCYAP1, KRTAP21-1, CCNA2, ARHGAP27, BUD31, GNAQ, HTR7, PSMG2, CDKN2B, CENPF, CCNA2, CCNE2, FAM58A, EPGN, TGFA, ANAPC10, EGF, ZFP91, ADAM10, CNTF, TAF9B, AMOT, RNF6, NOP58, MTOR, DCLK1, SERTAD2, DRD2, RICTOR, RBP4, SOCS2, MSTN, NPY1R, IGSF11, HTRA1, PSMG2, CDKN2B, EPGN, CENPF, TGFA, ANAPC10, EGF, CCNA2, HAUS3, MPHOSPH9, HAUS6, CDKN2B, NEK1, SUN2, CENPF, NEK9, HORMAD1, ITGB1, CCNE2, BBS4, HAUS3, RBP4, PARD6B, HAUS6, NEK1, CENPF, NEK9, PELO, ANAPC10, CCNA2, MPHOSPH9, HORMAD1, MPHOSPH9, SUN2, FBXO43, HORMAD1, DMC1, HMG20B, BANP, VASH1, CDKN2B, FANCI, ITGB1, CDKN2B, IL8, VASH1, PKD2, PKD1, FANCI, IL8, HMG20B, BANP, VASH1,	16.77
INTERPRO	IPR001336:EGF, type 1	3	0.017		14.34
GOTERM_BP_FAT	GO:0010647~positive regulation of cell communication	20	0.027		1.70
GOTERM_BP_FAT	GO:0010638~positive regulation of organelle organization	8	0.029		2.69
GOTERM_BP_FAT	GO:0051783~regulation of nuclear division	6	0.049		2.99
GOTERM_BP_FAT	GO:0007088~regulation of mitosis	6	0.049		2.99
GOTERM_BP_FAT	GO:0045742~positive regulation of epidermal growth factor receptor signaling pathway	3	0.057		7.62
GOTERM_BP_FAT	GO:0009967~positive regulation of signal transduction	17	0.064		1.61
GOTERM_MF_FAT	GO:0005154~epidermal growth factor receptor binding	3	0.089		5.92
GOTERM_BP_FAT	GO:0007176~regulation of epidermal growth factor receptor activity	3	0.098		5.59
GOTERM_BP_FAT	GO:0010564~regulation of cell cycle process	8	0.113		1.96
GOTERM_BP_FAT	GO:0010469~regulation of receptor activity	3	0.134		4.66
GOTERM_BP_FAT	GO:0043406~positive regulation of MAP kinase activity	7	0.157		1.92
GOTERM_BP_FAT	GO:0001525~angiogenesis	9	0.160		1.70
GOTERM_BP_FAT	GO:0042325~regulation of phosphorylation	22	0.162		1.32
GOTERM_BP_FAT	GO:0000187~activation of MAPK activity	6	0.169		2.05
GOTERM_BP_FAT	GO:0007243~protein kinase cascade	18	0.173		1.36
GOTERM_BP_FAT	GO:0010627~regulation of protein kinase cascade	13	0.183		1.46
GOTERM_BP_FAT	GO:0030071~regulation of mitotic metaphase/anaphase transition	3	0.184		3.81
GOTERM_BP_FAT	GO:0042058~regulation of epidermal growth factor receptor signaling pathway	3	0.198		3.65
GOTERM_BP_FAT	GO:0051174~regulation of phosphorus metabolic process	22	0.212		1.27
GOTERM_BP_FAT	GO:0019220~regulation of phosphate metabolic process	22	0.212		1.27
GOTERM_BP_FAT	GO:0051785~positive regulation of nuclear division	3	0.224		3.35
GOTERM_BP_FAT	GO:0045840~positive regulation of mitosis	3	0.224		3.35
GOTERM_BP_FAT	GO:0043405~regulation of MAP kinase activity	8	0.239		1.59
GOTERM_BP_FAT	GO:0045859~regulation of protein kinase activity	16	0.253		1.30
GOTERM_BP_FAT	GO:0045860~positive regulation of protein kinase activity	11	0.276		1.38
GOTERM_BP_FAT	GO:0051726~regulation of cell cycle	15	0.296		1.27
GOTERM_BP_FAT	GO:0043549~regulation of kinase activity	16	0.297		1.25
GOTERM_BP_FAT	GO:0007346~regulation of mitotic cell cycle	8	0.300		1.47
GOTERM_BP_FAT	GO:0033674~positive regulation of kinase activity	11	0.312		1.33
GOTERM_BP_FAT	GO:0000165~MAPKKK cascade	9	0.336		1.37
GOTERM_BP_FAT	GO:0051338~regulation of transferase activity	16	0.352		1.20
GOTERM_BP_FAT	GO:0051347~positive regulation of transferase activity	11	0.355		1.28
GOTERM_BP_FAT	GO:0043085~positive regulation of catalytic activity	20	0.500		1.08
GOTERM_BP_FAT	GO:0044093~positive regulation of molecular function	22	0.523		1.05
GOTERM_BP_FAT	GO:0032147~activation of protein kinase activity	5	0.585		1.23
GOTERM_BP_FAT	GO:0045787~positive regulation of cell cycle	3	0.609		1.47
GOTERM_BP_FAT	GO:0007093~mitotic cell cycle checkpoint	4	0.198		2.60
SP_PIR_KEYWORDS	cyclin	4	0.253		2.29
GOTERM_BP_FAT	GO:0007346~regulation of mitotic cell cycle	8	0.300		1.47
GOTERM_BP_FAT	GO:0000075~cell cycle checkpoint	5	0.410		1.54
KEGG_PATHWAY	hsa04110:Cell cycle	4	0.848		0.86
GOTERM_BP_FAT	GO:0045793~positive regulation of cell size	4	0.245		2.33
GOTERM_BP_FAT	GO:0030307~positive regulation of cell growth	3	0.445		2.00
GOTERM_BP_FAT	GO:0008361~regulation of cell size	9	0.455		1.22
GOTERM_BP_FAT	GO:0045927~positive regulation of growth	4	0.548		1.40

GOTERM_BP_FAT	GO:0032535~regulation of cellular component size	10	0.635		1.03
GOTERM_BP_FAT	GO:0040008~regulation of growth	12	0.678		0.98
GOTERM_BP_FAT	GO:0001558~regulation of cell growth	7	0.696		1.01
GOTERM_BP_FAT	GO:0007346~regulation of mitotic cell cycle	8	0.300		1.47
GOTERM_BP_FAT	GO:0051329~interphase of mitotic cell cycle	4	0.717		1.09
GOTERM_BP_FAT	GO:0051325~interphase	4	0.735		1.05
GOTERM_BP_FAT	GO:0000278~mitotic cell cycle	12	0.774		0.91
GOTERM_BP_FAT	GO:0051301~cell division	12	0.486		1.14
GOTERM_BP_FAT	GO:0000087~M phase of mitotic cell cycle	9	0.550		1.12
SP_PIR_KEYWORDS	cell division	10	0.575		1.08
SP_PIR_KEYWORDS	mitosis	7	0.619		1.09
GOTERM_BP_FAT	GO:0000279~M phase	12	0.631		1.02
SP_PIR_KEYWORDS	cell cycle	16	0.646		0.99
GOTERM_BP_FAT	GO:0007067~mitosis	8	0.675		1.02
GOTERM_BP_FAT	GO:0000280~nuclear division	8	0.675		1.02
GOTERM_BP_FAT	GO:0048285~organelle fission	8	0.714		0.98
GOTERM_BP_FAT	GO:0022403~cell cycle phase	14	0.722		0.95
GOTERM_BP_FAT	GO:0022402~cell cycle process	19	0.725		0.94
GOTERM_BP_FAT	GO:0000278~mitotic cell cycle	12	0.774		0.91
GOTERM_BP_FAT	GO:0007049~cell cycle	25	0.797		0.90
GOTERM_CC_FAT	GO:0005819~spindle	3	0.971		0.56

Annotation Cluster: JAK-STAT SIGNALING PATHWAY

Category	Term	Count	p Value	Genes	Fold Enrichment
GOTERM_BP_FAT	GO:0050730~regulation of peptidyl-tyrosine phosphorylation	8	0.010	FCER1A, ZFP91, IL5, CNTF, PDGFA, MTOR, RICTOR, EGF, IL22, ZFP91, CNTF, SPRY3, SOCS2,	3.29
GOTERM_BP_FAT	GO:0046425~regulation of JAK-STAT cascade	3	0.370		2.33
INTERPRO	IPR012351:Four-helical cytokine, core	3	0.479		1.87
KEGG_PATHWAY	hsa04630:Jak-STAT signaling pathway	5	0.833		0.87

Annotation Cluster: POSITIVE REGULATION OF ADENILATE CYCLASE ACTIVITY

Category	Term	Count	p Value	Genes	Fold Enrichment
GOTERM_BP_FAT	GO:0030814~regulation of cAMP metabolic process	8	0.075	GNAQ, DRD2, HTR7, GRM7, NF1, PKD2, NPY1R, ADCYAP1, RXFP1, PRKACB, PTGS2, EGF, TRAT1, MTNR1A, PDGFA, TUBAL3, HIST4H4, IL8, PIK3C2A, BUD31, HIST1H4A, HIST1H4J, NMU, CYSLTR1, PTH2R, TSHB, GRIN3A, ATP2B1, CYSLTR1, PLN, FABP4, ANAPC10, FABP4, ANAPC10,	2.17
GOTERM_BP_FAT	GO:0030799~regulation of cyclic nucleotide metabolic process	8	0.110		1.98
GOTERM_BP_FAT	GO:0006140~regulation of nucleotide metabolic process	8	0.121		1.93
GOTERM_BP_FAT	GO:0045761~regulation of adenylate cyclase activity	7	0.129		2.04
GOTERM_BP_FAT	GO:0031279~regulation of cyclase activity	7	0.143		1.98
GOTERM_BP_FAT	GO:0030817~regulation of cAMP biosynthetic process	7	0.152		1.94
GOTERM_BP_FAT	GO:0051339~regulation of lyase activity	7	0.152		1.94
GOTERM_BP_FAT	GO:0007188~G-protein signaling, coupled to cAMP nucleotide second messenger	6	0.187		1.97
GOTERM_BP_FAT	GO:0030808~regulation of nucleotide biosynthetic process	7	0.200		1.78
GOTERM_BP_FAT	GO:0030802~regulation of cyclic nucleotide biosynthetic process	7	0.200		1.78
GOTERM_BP_FAT	GO:0051051~negative regulation of transport	8	0.208		1.66
GOTERM_BP_FAT	GO:0007187~G-protein signaling, coupled to cyclic nucleotide second messenger	7	0.228		1.70
KEGG_PATHWAY	hsa04540:Gap junction	6	0.232		1.81
GOTERM_BP_FAT	GO:0019933~cAMP-mediated signaling	6	0.252		1.77
GOTERM_BP_FAT	GO:0051048~negative regulation of secretion	4	0.313		2.03
GOTERM_BP_FAT	GO:0019935~cyclic-nucleotide-mediated signaling	7	0.320		1.51
GOTERM_BP_FAT	GO:0045762~positive regulation of adenylate cyclase activity	4	0.323		2.00
GOTERM_BP_FAT	GO:0019932~second-messenger-mediated signaling	11	0.331		1.31
GOTERM_BP_FAT	GO:0031281~positive regulation of cyclase activity	4	0.333		1.96
GOTERM_BP_FAT	GO:0051349~positive regulation of lyase activity	4	0.353		1.89

GOTERM_BP_FAT	GO:0007631~feeding behavior	4	0.449		1.62
GOTERM_BP_FAT	GO:0007623~circadian rhythm	3	0.493		1.82
GOTERM_BP_FAT	GO:0031280~negative regulation of cyclase activity	3	0.589		1.52
GOTERM_BP_FAT	GO:0051350~negative regulation of lyase activity	3	0.589		1.52
GOTERM_BP_FAT	GO:0007194~negative regulation of adenylate cyclase activity	3	0.589		1.52
GOTERM_BP_FAT	GO:0007190~activation of adenylate cyclase activity	3	0.589		1.52
KEGG_PATHWAY	hsa04080:Neuroactive ligand-receptor interaction	10	0.612		1.05
KEGG_PATHWAY	hsa04020:Calcium signaling pathway	6	0.787		0.92
GOTERM_BP_FAT	GO:0043086~negative regulation of catalytic activity	6	0.972		0.61
GOTERM_BP_FAT	GO:0044092~negative regulation of molecular function	7	0.981		0.59

Annotation Cluster: **CYCLINS**

Category	Term	Count	p Value	Genes	Fold Enrichment
SP_PIR_KEYWORDS	cyclin	4	0.253	CCNE2, CDKN2B, FAM58A, CCNA2	2.29
INTERPRO	IPR006671:Cyclin, N-terminal	3	0.320		2.61
INTERPRO	IPR013763:Cyclin-related	3	0.371		2.32
SMART	SM00385:CYCLIN	3	0.396		2.20
INTERPRO	IPR006670:Cyclin	3	0.408		2.15

Annotation Cluster: **FATTY ACID METABOLIC PROCESS**

Category	Term	Count	p Value	Genes	Fold Enrichment
GOTERM_BP_FAT	GO:0046320~regulation of fatty acid oxidation	3	0.264	CAB39L, MTOR, PPARGC1A, BBS4, PDGFA, ACER1,	2.99
GOTERM_BP_FAT	GO:0019216~regulation of lipid metabolic process	6	0.371		1.50
GOTERM_BP_FAT	GO:0019217~regulation of fatty acid metabolic process	3	0.527		1.71
GOTERM_BP_FAT	GO:0010565~regulation of cellular ketone metabolic process	3	0.609		1.47

Annotation Cluster: **HOMEOBOX**

Category	Term	Count	p Value	Genes	Fold Enrichment
UP_SEQ_FEATURE	DNA-binding region:Homeobox	7	0.661	HESX1, BARX2, LMX1B, HOXA10, PAX3, POU1F1, HDX, VSX1, POGZ, EPAS1, RXRA, MBD2, PRDM16, FOXP1, TAL1, NR2F6, SPIC, NFIL3,	1.05
SMART	SM00389:HOX	8	0.691		1.00
INTERPRO	IPR017970:Homeobox, conserved site	8	0.702		0.99
INTERPRO	IPR001356:Homeobox	8	0.715		0.98
INTERPRO	IPR012287:Homeodomain-related	8	0.727		0.96
SP_PIR_KEYWORDS	Homeobox	8	0.744		0.95
GOTERM_MF_FAT	GO:0043565~sequence-specific DNA binding	17	0.929		0.77

Annotation Cluster: **TRANSCRIPTION FACTOR ACTIVITY**

Category	Term	Count	p Value	Genes	Fold Enrichment
SP_PIR_KEYWORDS	transcription regulation	71	0.554	ENY2, ZBTB33, CHURC1, ARID4A, ARNT2, NAA15, PAX3, MAF1, TMF1, TAF7L, KDM1B, GABPB1, ZFP91, BARX2, NR2F6, SPIC, NFIL3, KDM5B, SERTAD2, ZNF101, ELP4, SNAPC5, RREB1, YY1, RXRA, SOX11, ZFX, HDAC10, HMG20B, BANP, LPIN2, MBD2, PPARGC1A, ZNF335, FRY, SUZ12, ZFP82, BAZ1B, RFC1, MLLT10, MNDA, KRTAP1-1, PRDM2, WASL, VOPP1, TFAP2E, ASCL4, CRTC3, SUPT3H, TAF9B, ZNF511, PRDM16, POU1F1, CCDC59, MED12L, ZNF32, HESX1, TAL1, CIR1, HOXA10, CHD1, RUNX1, SLC30A9, AEBP2, ZMYM2, EPAS1, LMX1B, KLF15, VSX1, FOXP1, CNTF, TBL1Y, ADH1A, HDX, BUD31, RNF6, FABP4, DZIP1, HIST1H2BL, INTS6, PRKACB, CCNA2, C15ORF55, MTUS1, DHX32, CIZ1, H2AFJ, RPS19, CELF4, NEK9, PELO, BIVM, HMGB1, HDLBP, LMNB1, NFKBID, NEK1, CHX, TTC5, LIN28B, HIST1H4A, POLN, HIST1H4J, NXF5, HEATR1, NOP10, HN1L, PPIH, MSL1, HIST1H3A, CPNE1, HIST1H3H, PLEKHA2, HIST4H4, SHOC2, GNL3L, CCNE2, DCAF13, DKC1, FANCI, IPMK, POGZ, HIST1H1C, PIK3C2A, HIST1H1A, G3BP1, LARP7, RIPPLY2, DMC1, UTP3, UTP6, TSPYL1, DCLK3, DAZL, RSL24D1, HIST1H2BC, DAZ2, SUN2, CENPF, SYNPO2, SNAI1, RSBN1, DIS3, RPS6KA5, RNF43, PSMG2, HIST1H2AI, HSPA4L, SH3BGRL2, NOP58, SH3D19, SSNA1, TLR3, IL22, TARBP1, ACVR2B, REM2, IL5, MSTN, EDA2R, SFMBT1, IL22,	1.00
GOTERM_MF_FAT	GO:0030528~transcription regulator activity	54	0.616		0.99
SP_PIR_KEYWORDS	Transcription	71	0.631		0.98
SP_PIR_KEYWORDS	nucleus	143	0.771		0.96
GOTERM_MF_FAT	GO:0003700~transcription factor activity	32	0.798		0.91
GOTERM_BP_FAT	GO:0045449~regulation of transcription	87	0.806		0.93
GOTERM_BP_FAT	GO:0006355~regulation of transcription, DNA-dependent	57	0.859		0.90
GOTERM_BP_FAT	GO:0051252~regulation of RNA metabolic process	58	0.870		0.89
GOTERM_BP_FAT	GO:0006350~transcription	67	0.890		0.89
SP_PIR_KEYWORDS	dna-binding	56	0.924		0.86
GOTERM_MF_FAT	GO:0003677~DNA binding	70	0.975		0.83
